# Using Genealogical Concordance and Coalescent-Based Species Delimitation to Assess Species Boundaries in the *Diaporthe eres* Complex

**DOI:** 10.3390/jof7070507

**Published:** 2021-06-25

**Authors:** Sandra Hilário, Micael F. M. Gonçalves, Artur Alves

**Affiliations:** Centre for Environmental and Marine Studies (CESAM), Department of Biology, Campus Universitário de Santiago, University of Aveiro, 3810-193 Aveiro, Portugal; sandra.hilario@ua.pt (S.H.); mfmg@ua.pt (M.F.M.G.)

**Keywords:** GCPSR, PTP, species delimitation, taxonomy

## Abstract

DNA sequence analysis has been of the utmost importance to delimit species boundaries in the genus *Diaporthe*. However, the common practice of combining multiple genes, without applying the genealogical concordance criterion has complicated the robust delimitation of species, given that phylogenetic incongruence between loci has been disregarded. Despite the several attempts to delineate the species boundaries in the *D. eres* complex, the phylogenetic limits within this complex remain unclear. In order to bridge this gap, we employed the Genealogical Phylogenetic Species Recognition principle (GCPSR) and the coalescent-based model Poisson Tree Processes (PTPs) and evaluated the presence of recombination within the *D. eres* complex. Based on the GCPSR principle, presence of incongruence between individual gene genealogies, i.e., conflicting nodes and branches lacking phylogenetic support, was evident. Moreover, the results of the coalescent model identified *D. eres* complex as a single species, which was not consistent with the current large number of species within the complex recognized in phylogenetic analyses. The absence of reproductive isolation and barriers to gene flow as well as the high haplotype and low nucleotide diversity indices within the above-mentioned complex suggest that *D. eres* constitutes a population rather than different lineages. Therefore, we argue that a cohesive approach comprising genealogical concordance criteria and methods to detect recombination must be implemented in future studies to circumscribe species in the genus *Diaporthe*.

## 1. Introduction

A reliable and accurate identification of fungal plant pathogens is of the utmost importance in disease diagnosis to implement effective management and quarantine strategies [1]. However, one crucial aspect in recognizing a fungal species is correctly defining a species [2]. Although molecular approaches have changed our perception of fungal diversity [3], difficulties in understanding the evolutionary processes in fungi have arisen, thus turning a correct definition of a fungal species into a prevailing challenge to mycologists [4,5].

The Genealogical Concordance Phylogenetic Species Recognition (GCPSR), introduced by Taylor et al. [6], relies in the comparison of individual gene genealogies to identify incongruences, and it has been particularly useful to delimit the species boundaries in morphologically conserved fungi [7]. However, the common approach of concatenating sequence data to delimit species without following the GCPSR principle [6,8] overestimates the true diversity of species, since each clade in combined trees is frequently recognized as a distinct lineage [9,10]. Moreover, species boundaries in closely related taxa can be somehow difficult to determine through multilocus sequence data, as some alleles are not expected to be reciprocally monophyletic in the initial stages of speciation, resulting in phylogenetic incongruences [7,11]. As an alternative, the delimitation of species using multi-species coalescent models provides a more comprehensive insight into the speciation events, as it can estimate species boundaries even in the presence of incongruence between individual genealogies [12].

Some authors have already advocated that the description of fungal species needs to be based on approaches that employ a combination of several methods: phylogenetic analysis following the genealogical concordance (GCPSR) [6], coalescence models such as Poisson Tree Processes (PTPs) [13], and splits graphs (Phylogenetic networks) [2]. Nevertheless, despite the usefulness of the above-mentioned methods to support the boundaries of species, there are only a few studies on fungi, namely in *Colletotrichum* [7], *Beauveria* [14] and more recently, *Diaporthe* [15].

*Diaporthe* species are associated with a wide range of plant hosts as pathogens, endophytes or saprobes of crops, ornamentals, and forest trees [16,17,18]. Species identification in the genus *Diaporthe* has evolved from host association and morphology [19,20] to the widespread adoption of DNA sequencing [21]. Currently, the nuclear ribosomal internal transcribed spacer (ITS), the translation elongation factor 1-α (*TEF1-α*), beta-tubulin (*TUB2*), histone H3 (*HIS3*), and calmodulin (*CAL*) genes are the most used molecular loci in this genus, which currently includes the description of over 200 species supported by ex-type cultures and supplementary DNA sequences [4,22].

*Diaporthe eres* is the type species of the genus and was originally described by Nitschke (1870), from *Ulmus* sp. in Germany [23]. In 1933, Wehmeyer listed a number of synonyms under *D. eres* based on morphological characters. Later, based on molecular data, few of these synonyms were accepted by several authors [21,23]. Udayanga et al. [23], attempted to define the species limits of *D. eres* and closely related species based on the concatenation of seven loci and designated an epitype specimen for *D. eres*. Despite the attempts by several authors to re-examine the *D. eres* complex, its boundaries are not entirely understood [17,18,24]. In several studies, many species have been demonstrated to be synonymous to the *D. eres* species, based on multi-locus analyses such us *D. biguttusis*, *D. camptothecicola*, *D. castaneae-mollissimae*, *D. cotoneastri*, *D. ellipicola*, *D. longicicola*, *D. mahothocarpus*, *D. momicola* and *Phomopsis fukushii* [18,23,24]. In a recent study, Chaisiri et al. [25] found that *D. henanensis*, *D. lonicerae* and *D. rosicola* were also synonyms of *D. eres*, based on the GCPSR principle coupled with haplotype network analysis and population genetic diversity. Although *D. eres* has been regarded as a minor pathogen, it is also considered one of the main causal agents of cankers on grapevines [17], blueberries [26] and apple trees [27]. Lopes et al. [28] reported the occurrence of *D. eres* in forest trees but found some difficulties in the interpretation of their phylogenetic analyses and delimitation of the species in the *D. eres* complex. Therefore, the aim of this study was to clarify the limits of the *D. eres* species complex by implementing several distinct methods, such as single and multilocus phylogenetic analyses, the genealogical concordance principle, coalescent-based species delimitation methods (PTP), phylogenetic networks, pairwise homoplasy index test and comparison of morphological characters.

## 2. Materials and Methods

### 2.1. Fungal Isolates

In 2007, a survey was conducted to inspect for the presence of *Diaporthe* species associated with ornamental plants in Portugal. Twigs and leaves of *Banksia* sp. showing blight symptoms typical of *Diaporthe* were collected. Fungal isolates were obtained by the methods described by Hilário et al. [29], from which a fungus was collected that resembled *D. eres*. Moreover, isolates CAA954 and CAA1001 obtained previously from *Quercus suber* and *Pinus pinaster* and identified as *D. eres* [28] were also included in this study. Cultures were maintained in the collection of Artur Alves (CAA), University of Aveiro (Portugal), on potato dextrose agar (PDA) (Merck, Darmstadt, DE, Germany). The isolates used in this study are listed in Table 1.

### 2.2. DNA Extraction and PCR Amplification

The genomic DNA was extracted from 7-day-old fungal isolates, grown at 25 °C, according to a modified protocol of Möller [30]. The nuclear ribosomal internal transcribed spacer region using the primers set ITS5 and NL4 [31] was amplified as described by Alves et al. [32]. The primers set EF-688F/EF-1251R [33], T1/Bt2b [34,35], CYLH3F/H3-1bR [34,36] and CAL-38F/CAL-737R [29,37] were used to amplify part of *TEF1-α*, *TUB*2, *HIS3* and *CAL* loci, respectively.

All PCR reaction mixtures, with a final volume of 25 µL, were composed of 15.75 µL of sterile pure water, 6.25 µL of NZYTaq 2xgreen Master Mix (Nzytech^TM^, Lisbon, PT, Portugal), 1 µL of each primer at 10 pmol/µL and 1 µL of DNA template. PCR reactions were performed as described by Hilário et al. [28]. The amplified PCR fragments were purified using the NZYGelPure Kit (Nzytech^TM^, Lisbon, Portugal) and sequenced by GATC Biotech (Cologne, DE, Germany).

### 2.3. Phylogenetic Analyses

Multilocus phylogenetic analyses based on 5 loci (ITS, *TEF1-α*, *TUB2*, *HIS3* and *CAL*) and 4 loci (*TEF1-α*, *TUB2*, *HIS3* and *CAL*) were performed for the *Diaporthe eres* species complex. The alignments of different gene regions, including sequences of the isolates used in this study and those retrieved from GenBank (Table 1), were performed with ClustalX2.1 software [38] using the following parameters: pairwise alignment (gap opening = 10, gap extension = 0.1) and multiple alignment (gap opening = 10, gap extension = 0.2, transition weight = 0.5, delay divergent sequences = 25%). The alignments were checked and edited manually with BioEdit Alignment Editor v.7.0.5.3 [39] and concatenated using Sequence Matrix software [40]. Phylogenetic analyses of sequence data were done using PAUP* v.4.0b10 [41] for Maximum Parsimony (MP) analyses, MrBayes v.3.0b4 [42] for Bayesian Inference (BI) analyses and MEGA v.7 [43] for Maximum Likelihood (ML) analyses. Trees resulting from the MP and BI analyses were visualized with TreeView [44]. Maximum Parsimony analyses were performed using the heuristic search option with 100 random taxon additions and subtree pruning regrafting (SPR) method as the branch-swapping algorithm. All characters were unordered and of equal weight, and gaps were treated as missing data. Maxtrees were set to 100 and branches of zero length were collapsed. Clade stability was assessed using a bootstrap analysis with 1000 replicates. The parameters including consistency index (CI), retention index (RI), tree length (TL), rescaled consistency index (RC) and homoplasy index (HI) were also calculated. Bayesian analyses employing a Markov Chain Monte Carlo sampling (MCMC) method were performed. The general time-reversible model of evolution [45], including estimation of invariable sites and assuming a gamma distribution with six rate categories was used for BI analyses. Four MCMC chains were run simultaneously, starting from random trees for 1,000,000 generations, and sampled every 100th generation for a total of 10,000 trees. The first 1000 were discarded as the burn-in phase of each analysis. Posterior probabilities (PPs) were determined from a majority-rule consensus tree generated with the remaining 9000 trees. Maximum likelihood analyses were performed on a Neighbor-Joining starting tree automatically generated by the software. Nearest-Neighbor-Interchange (NNI) was used as the heuristic method for tree inference, and 1000 bootstrap replicates were performed. MEGA v.7 was also used to determine the best nucleotide substitution model to be used for building the ML trees. The phylogenetic analyses included six well-delimitated *Diaporthe* species. To examine the possibility of a combined dataset, sequences from the five and four loci were aligned and combined, and the Incongruence Length Difference test was conducted in PAUP* v.4.0b10 [41]. Moreover, the highly supported clades in individual genealogies were also compared in order to detect conflict among them [33]. Individual gene trees of the *D. eres* species complex, comprising all available species for each locus were also constructed. These trees were rooted to *Diaporthe citri* and *D. citrichinensis*. The sequence generated in this study was deposited in GenBank (Table 1) (www.ncbi.nlm.nih.gov, accessed on 30 August 2020). The phylogenetic tree and alignments were deposited in TreeBASE (www.TreeBASE.org; Study Number S27078, accessed on 13 October 2020).

### 2.4. Pairwise Homoplasy Index Test and Phylogenetic Network Analysis

The concatenated five loci tree was used to infer the occurrence of sexual recombination within the *D. eres* complex, through the pairwise homoplasy index test (PHI) [46] implemented in SplitsTree v.4.16.1 (www.splitstree.org, accessed on 10 November 2020) [47]. To detect intragenic recombination, individual loci were also analyzed using the PHI test. Significant recombination was considered with a PHI index below 0.05 (Φw < 0.05). The relationships between closely related taxa were visualized by constructing a phylogenetic network from the concatenated dataset of five loci, using the LogDet transformation and the NeighborNet algorithm options implemented in SplitsTree v.4.16.1 [47].

### 2.5. Species Delimitation Analyses

To infer the species boundaries of the *Diaporthe eres* complex, coalescence-based methods were performed based on the combined alignment of ITS, *TEF1-α*, *TUB2*, *HIS3* and *CAL* genes. First, the single Poisson Tree Processes model (PTP) [13], was used. The newick-format tree produced by MEGA v.7 was used for the PTP analysis with the following parameters: 500,000 MCMC generations, thinning set to 100, burn-in at 50,000 generations and conducted on the web server for PTP (http://species.h-its.org/ptp/, accessed on 24 November 2020). Second, the multi-rate PTP (mPTP), which can accommodate data sets comprised of species with different levels of molecular diversity [48], was also conducted on the web server for mPTP (http://mptp.h-its.org, accessed on 24 November 2020), using the same newick-format tree as for the single PTP analysis.

### 2.6. Population Genetic Diversity

To study the genetic diversity within the *Diaporthe eres* complex, diversity indices were calculated for each gene region and the combined datasets. The pairwise nucleotide diversity (π), haplotype diversity (hd), number of haplotypes (h), number of polymorphic sites (S) and the Tajima’s D statistical test [49] were calculated on DnaSP program v.6.12 (Barcelona, Spain) [50]. These parameters were calculated from a combined dataset of 19 ex-type species and 21 taxonomically authenticated isolates belonging to the *D. eres* complex (Table 1).

### 2.7. Morphology of the Diaporthe eres Species Complex

The alpha conidia, beta conidia and conidiophores length/width (L/W) ratios of all current species belonging to the *Diaporthe eres* complex were calculated. A hierarchical clustering analysis using the Ward’s method [51] was carried out, and a dendrogram was generated in R Statistical Software v.4.0.2 [52] using the _DENDEXTEND_ package [53].

## 3. Results

### 3.1. Phylogenetic Analyses and Informative Characters

The individual gene trees of the complex (Figures S1–S5) showed that the isolates used in this study cluster in a clade containing 29 species, designated here as the *D. eres* species complex. The partition homogeneity test for both 5-loci and 4-loci combined alignments resulted in a low *p*-value (*p* = 0.01), indicating that the genes are unsuitable to be combined. Nevertheless, despite the observed incongruences, multilocus analyses were constructed based on five and four loci. The five loci combined alignment comprises 2364 characters (537 characters from ITS, 387 from *TEF1-α*, 430 from *TUB2*, 522 from *CAL*, and 488 from *HIS3*, including alignment gaps and indel coding); the four loci combined alignment is comprised of 1827 characters (387 from *TEF1-α*, 430 from *TUB2*, 522 from *CAL*, and 488 from *HIS3*, including alignment gaps and indel coding). Both analyses included 12 well-delimitated outgroup taxa and 40 ingroup taxa (3 from this study and 37 taxa retrieved from GenBank) (Table 1). The parsimony informative characters and the nucleotide models used for each analysis are summarized in Table 2. Moreover, sequences of the five genes were aligned and analyzed separately by Maximum Likelihood, Maximum Parsimony and Bayesian Inference analyses, and the resulting trees were compared. Only ML single trees are shown with bootstrap and posterior probabilities given for those well-supported clades (Figures S6–S10). The ML, MP and BI analyses resulted in trees that were topologically similar.

According to the informative characters provided by the Maximum Parsimony analyses, *TEF1-a* displayed the most informative sequences, followed by *CAL*, *TUB2* and *HIS3* loci. ITS presented the lowest percentage, indicating that this locus is unreliable for the delimitation of the *D. eres* species. The combined four loci dataset (*TEF1-a*, *TUB2*, *CAL*, and *HIS3*) showed better delimitation for *D. eres* compared to the five loci dataset (Table 2), confirming that the ITS locus is not the appropriate locus to delineate species in this genus.

### 3.2. Species Delimitation Based on the GCPSR Principle

**Figure 1:**
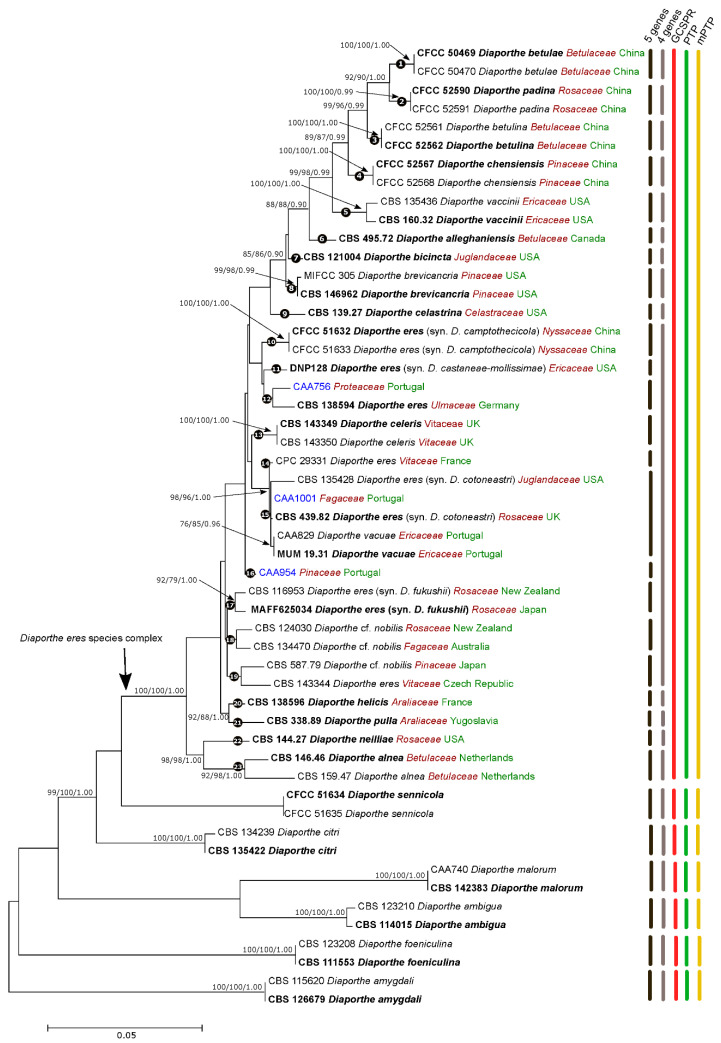
Maximum Likelihood (ML) tree of the *Diaporthe eres* complex and related species, based on ITS *TEF1-α*, *TUB*2, *HIS3* and *CAL* loci. The ML tree is drawn to scale and was constructed based on the Tamura-Nei parameter model, assuming a gamma distribution. ML and MP bootstrap values greater than 70% and posterior probabilities (PPs) greater than 0.80 are shown at the nodes. The ex-type strains are in bold. The isolates from this study are indicated in blue. Species name is followed by the family of the host it was isolated from (red) and country of origin (green).

The isolates from this study clustered in a highly supported clade on both 5-loci (ML/MP/PP = 100/100/1.00) (Figure 1) and 4-loci (ML/MP/PP = 99/99/1.00) (Figure S11) phylogenies. To assess species boundaries in the *D. eres* complex, the GCPSR principle was applied. Our results revealed conflicts between individual phylogenies, with several nodes lacking phylogenetic support. For example, *D. vaccinii* is closely related to *D. celeris* in the *TUB2* tree (Figure S8), but it is a sister species to *D. camptothecicola* (previously synonymized with *D. eres*) in the ITS individual phylogram (Figure S6). Moreover, a lack of high bootstrap and posterior probability values on both individual and combined trees in several branches were observed, revealing poor phylogenetic support among the species. It is also evident that isolates from the same species cluster in different clades in the same individual gene tree. For instance, two isolates of *D. alnea* are phylogenetically distant in the *TUB2* tree (Figure S8), while they group together in the remaining individual phylograms. The same seems to occur with some isolates of *D. eres*: the isolates CBS 116953 and MAFF625034 (formerly described as *D. fukushii*) and two isolates of *D. camptothecicola*, previously synonymized with *D. eres*, do not cluster together in the ITS single locus tree (Figure S6). It is also notable from our analyses that the ex-type of *D. alnea* (CBS 146.46) is a close relative of *D. allenghaniensis* in the *TUB2* tree (Figure S8), whereas they are different taxa in the remaining individual and combined trees. Moreover, the species *D. brevicancria* is phylogenetically indistinguishable from *D. celastrina* and *D. maritima* in the individual *CAL* tree (Figure S10). Thus, by implementing the GCPSR principle, based on the comparison of more than one gene genealogy to identify phylogenetic concordance, we verified that the node delimiting the transition from concordant branches to incongruence corresponds to the *D. eres* complex (Figure 1). As estimated by the initial individual trees (Figures S1–S5), the species *D. maritima*, *D. phragmitis* and *D. rosicola* belong to the *D. eres* species complex. However, given the lack of some sequences for these species, they were not included in the 4-loci and 5-loci phylogenetic analyses. However, by analyzing the individual gene trees, and given their position within the complex, we advocate that the aforementioned species should also be assigned to *D. eres*. Contrarily, individual gene trees are concordant regarding the six well-delimited species (*D. citri*, *D. sennicola*, *D. malorum*, *D. foeniculina*, *D. ambigua* and *D. amygdali*), indicating that there are no conflicts among the individual phylograms. This provides solid evidence that these clades represent different species.

### 3.3. Species Delimitation Based on Poisson Tree Processes

**Figure 2:**
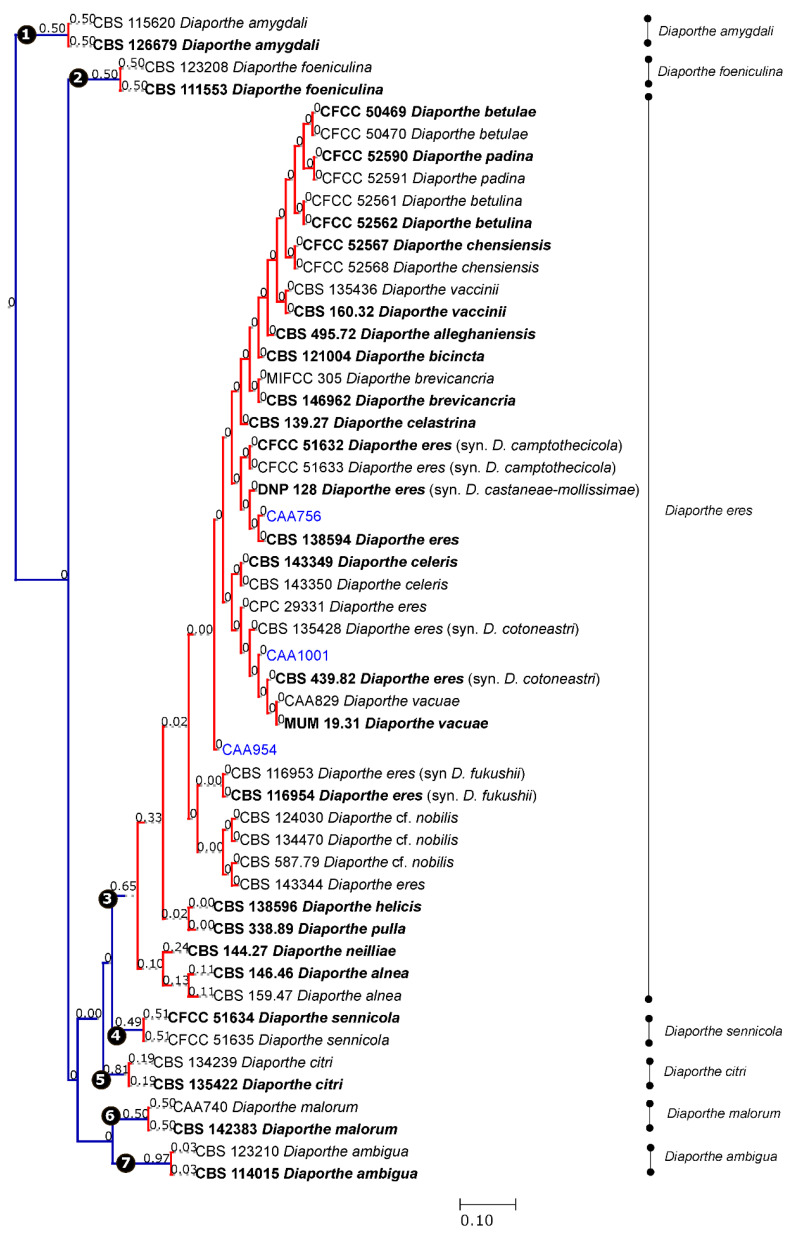
Results of the single PTP analyses for the *Diaporthe eres* species complex and related taxa, based on Bayesian and Maximum Likelihood topologies. Putative species clusters are indicated using transitions from blue-colored to red-colored branches and represented by circles (1–7). The isolates obtained in this study are indicated in blue.

**Figure 3:**
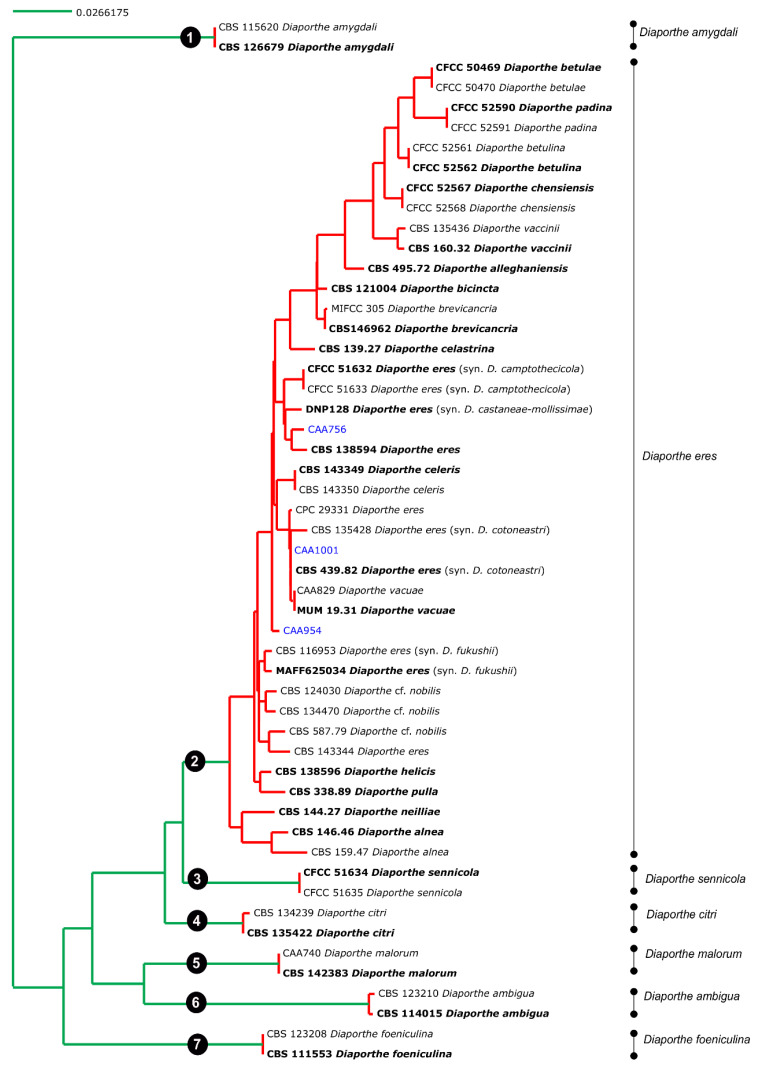
Results of the mPTP analysis for the *Diaporthe eres* species complex and related taxa. Putative species clusters are indicated using transitions from green-colored to red-colored branches. The isolates obtained in this study are indicated in blue.

Given the lack of *TEF1-α*, *CAL* and *HIS3* sequences for some species of the complex, the coalescent model applied included only those species whose five loci were available. Both single PTP and mPTP analyses gave congruent results and recognized the *D. eres* complex as a single species. From the analyses, the transition from blue-colored to red-colored branches (in single PTP, Figure 2), and the transition from green-colored to red-colored (in multi-rate PTP, Figure 3), evidenced that all species were comprised in one clade only, showing that the complex should be considered as a population rather than different taxa. In addition to this, a PTP analysis was performed based on the 4-loci combined alignment (Figure S12), excluding the ITS data, in order to understand whether the delimitation of species would be the same. This result corroborates the one from the PTP analysis based on the alignment of five genes, indicating that the ITS region did not affect the species delimitation, in contrast to the phylogenetic analysis. Therefore, we can consider that all species currently accepted in the *D. eres* complex should be recognized as a single species.

### 3.4. Pairwise Homoplasy Test and Phylogenetic Networks

**Figure 4:**
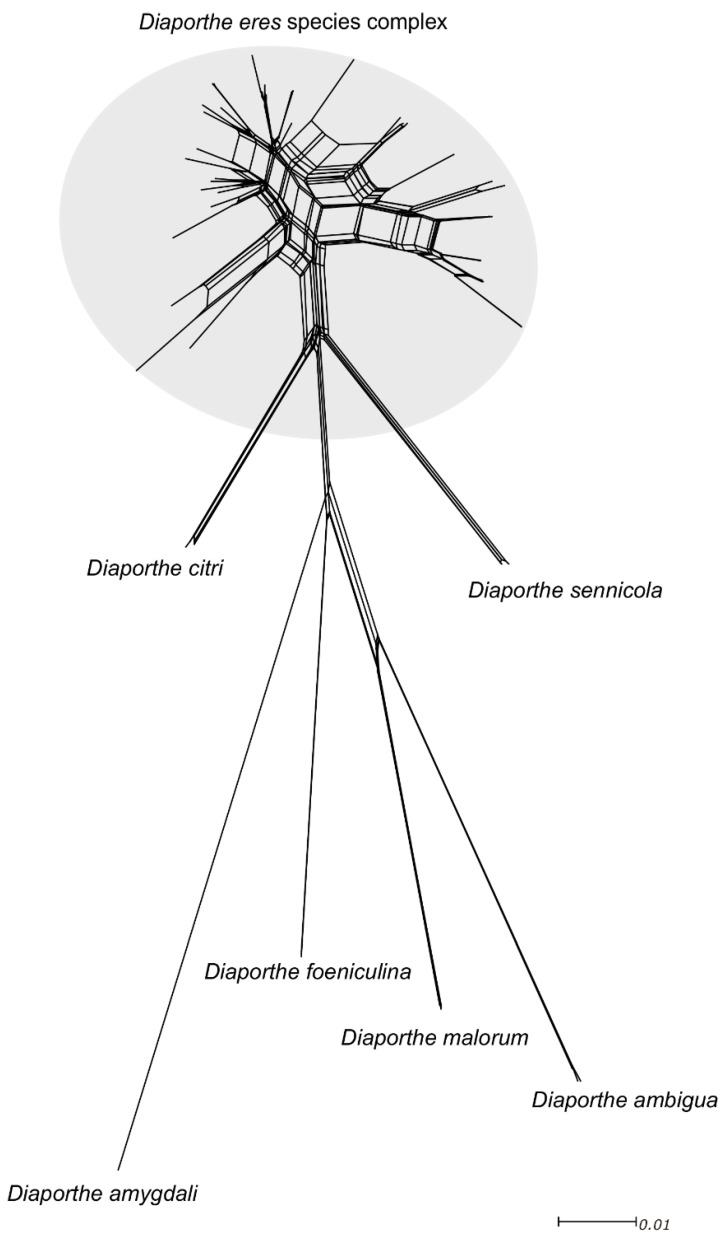
Phylogenetic network from the concatenated data (ITS, *TEF1-α*, *TUB2*, *HIS3* and *CAL*) representing the structure of the *Diaporthe eres* species complex and other well-delimitated species, based on the LogDet transformation and the NeighborNet algorithm, inferred by SplitsTree. The scale bar represents the expected number of substitutions per nucleotide position.

Significant recombination was detected within the *D. eres* complex when applying the PHI test (Φw = 0), denoting that there was no reproductive isolation within the group. Moreover, the pairwise homoplasy index test also confirmed that ITS and *TUB2* loci have a significant rate of recombination (Φw = 1.098 × 10^−7^ and Φw = 4.175 × 10^−6^, respectively). Subsequently, the network relationships in the *Diaporthe eres* complex (Figure 4) are shown with boxes, indicating the occurrence of recombination within the group. Additionally, based on the relative distance of species and structure of the phylogenetic network, all species in the *D. eres* complex should be regarded as one single species.

### 3.5. Population Genetic Diversity

The genetic diversity data for each gene region as well as for the combined dataset is summarized in Table 3. In this analysis, all loci showed a low number of nucleotide diversity (π = 0.009 to π = 0.025) and high values for all genes (hd = 0.882 to hd = 0.966). In addition to this, *TEF1-α*, *HIS3*, *TUB2*, *CAL* and the combined five and four loci alignments did not give statistically significant negative Tajima’s D values.

### 3.6. Morphology of the Diaporthe eres Species Complex

**Figure 5:**
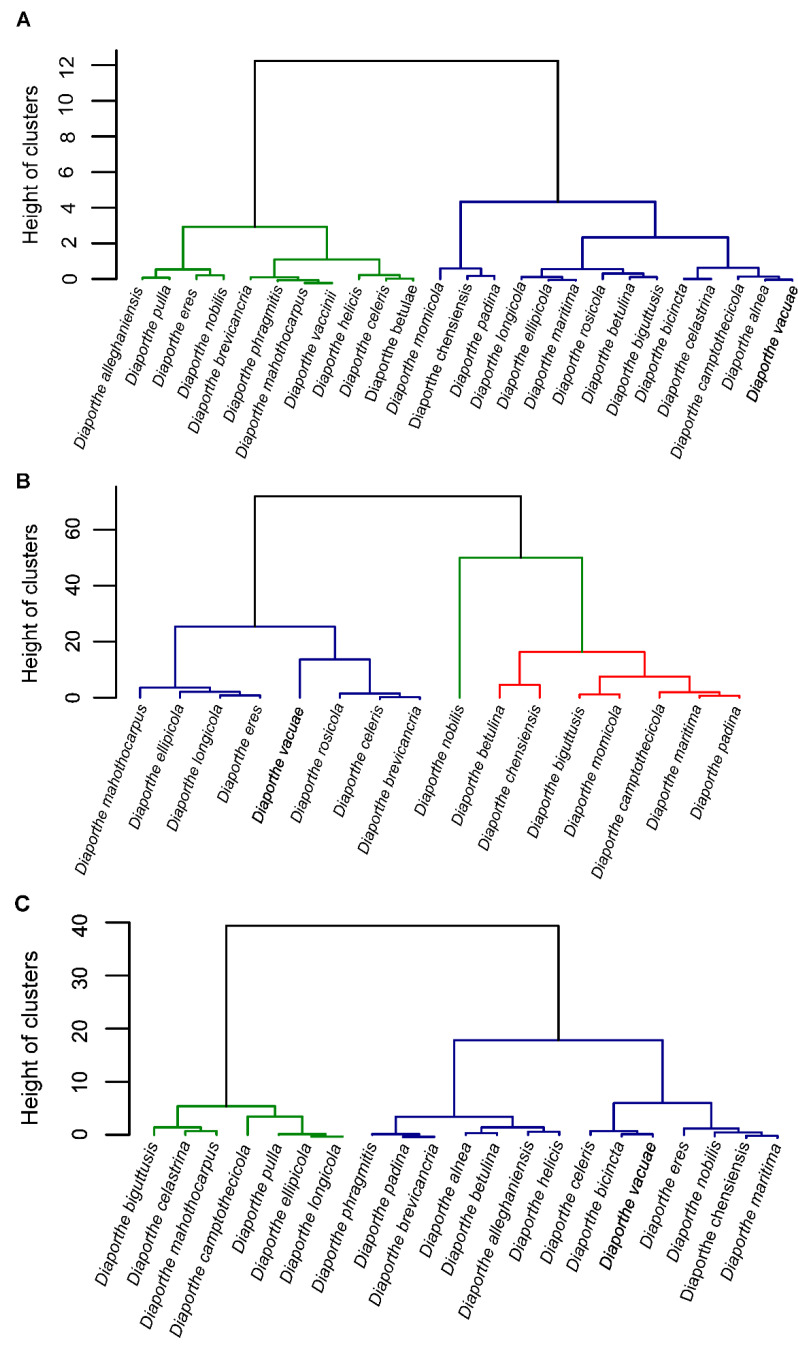
Dendrogram from the hierarchical clustering analysis based on the Ward’s method showing the distribution of the length and width ratio (L/W) of alpha conidia (**A**), beta conidia (**B**) and conidiophores (**C**) of all species isolates from the *Diaporthe eres* complex. Green, blue and red colors represent the different clusters based on the micromorphological descriptions.

Based on published morphological descriptions of 25 species belonging to the *D. eres* complex (excluding *D. neilliae*, which lacks a taxonomic description of the asexual morph), a hierarchical clustering analysis was performed using the Ward’s method. The dendrogram was constructed based on the ratios of alpha and beta conidia as well as conidiophores length and width (Figure 5).

## 4. Taxonomy

The present study combined phylogenetic analyses based on the genealogical concordance principle (GCPSR), coalescence (PTP and mPTP) and morphological comparisons to delimit the species boundaries in the *D. eres* complex. According to the aforementioned analyses, the *D. eres* complex constitutes a single species rather than different lineages. In order to compare and study the micromorphological characteristics of all these species a synopsis of conidiomata, conidiogenous cells and conidia characteristics are provided in Table 4.

*Diaporthe eres* Nitschke, Pyrenomycetes Germanici 2:245 (1870)
Basionym: *Phoma oblonga* Desm., Annales des Sciences Naturelles; Botanique, sér. 2, 22:218 (1853)
=*Diaporthe alnea* Fuckel, Jahrbücher des Nassauischen Vereins für Naturkunde 23–24:207 (1870)
=*Diaporthe pulla* Nitschke, Pyrenomycetes Germanici 2:249 (1870)
=*Diaporthe helicis* Niessl, Verhandlungen des naturforschenden Vereines in Brünn 16:50 (1876)
=*Diaporthe nobilis* Saccardo and Spegazzini, Michelia 1:386 (1878)
=*Diaporthe bicincta* (Cooke and Peck) Sacc., Sylloge fungorum (Abellini) 1:622 (1882)
=*Diaporthe neilliae* Peck, Annual Report on the New York State Museum of Natural History 39:52 (1887)
=*Diaporthe celastrina* (Ellis and Barthol), The Journal of Mycology 8:173 (1902)
≡*Phomopsis oblonga* (Desm.) Traverso, Fl. ital. crypt., Pars 1: Fungi. Pyrenomycetae. Xylariaceae, Valsaceae, Ceratostomataceae: 248 (1906)
=*Phomopsis velata* Sacc. Traverso, Fl. ital. crypt. (Florence) 2:248 (1906)
=*Diaporthe vaccinii* Shear, United States Department of Agriculture 258:7 (1931)
=*Diaporthe alleghaniensis* Arnold, Canadian Journal of Botany 45:787 (1967)
≡*Diaporthe cotoneastri* Udayanga, Crous and Hyde, Fungal Diversity 56:166 (2012)
≡*Diaporthe castaneae-mollisimae* Udayanga, Crous and Hyde, Fungal Diversity 56:166 (2012)
=*Diaporthe phragmitis* Crous, Fungal Planet 283:219 (2014)
=*Diaporthe biguttusis* Gao and Cai, Fungal Biology 119:300 (2015)
=*Diaporthe ellipicola* Gao and Cai, Fungal Biology 119:300 (2015)
=*Diaporthe longicicola* Gao and Cai, Fungal Biology 119:303 (2015)
=*Diaporthe mahothocarpus* Gao and Cai, Fungal Biology 119:306 (2015)
=*Diaporthe betulae* Tian and Fan, Phytotaxa 269:96 (2016)
=*Diaporthe maritima* Tanney, Fungal Biology 120:1454 (2016)
=*Diaporthe camptothecicola* Tian and Yang, Mycotaxon 132:595 (2017)
=*Diaporthe momicola* Dissanayake, Li and Hyde, Mycosphere 8:541 (2017)
=*Diaporthe fukushii* Dissanayake, Phillips and Hyde, Mycosphere 8:1130 (2017)
=*Diaporthe betulina* Tian and Yang, Mycokeys 39:121 (2017)
=*Diaporthe chensiensis* Tian and Yang, Mycokeys 39:127 (2017)
=*Diaporthe padina* Tian and Yang, Mycokeys 39:137 (2017)
=*Diaporthe celeris* Guarnaccia, Woodhall and Crous, Persoonia 40:146 (2018)
=*Diaporthe rosicola* Wanasinghe, Jones and Hyde, Fungal Diversity 89:187 (2018)
=*Diaporthe vacuae* Hilário, Santos and Alves, Mycologia 55:207 (2020)
=*Diaporthe brevicancria* Sakalidis and Medina-Mora, Phytopathology (2020)

Typification: as described by Udayanga et al. [23]—*Diaporthe eres*, Germany, Nordrhein-Westfalen, Munsterland, Munster Botanical Gardens, on twigs of *Ulmus* sp., June 1865, T. Nitschke, (lectotype B 70 0009145); Carpinion forest, on dead, attached, corticated twigs of *Ulmus laevis*, 5 January 2013, R. Jarling, comm. R. Schumacher (epitype BPI 892912, ex-epitype culture AR5193 = CBS 138594). *Phoma oblonga*, France, on twigs of *Ulmus campestris*, unknown collector (bound specimen of Desmazieres, Plantes Cryptogames du Nord de la France, Ed. 2, ser. 2. No. 60 in BPI). Germany, Carpinion forest, on dead, attached, corticated twigs of *Ulmus laevis*, 5 January 2013, R. Jarling, comm. R. Schumacher (epitype BPI 892913, ex-epitype culture AR5196 = CBS 138595). *Phomopsis castaneae-mollisimae*, China, Taian, Shangdong, leaf of *Castanea mollissima*, April 2006, S.X. Jiang (CLS 0612, holotype not seen, ex-type culture BYD1 = DNP128), ex-isotype culture BYD4 = DNP129. *Diaporthe cotoneastri*, UK, Scotland, Ayr, on *Cotoneaster* sp., May 1982, H. Butin (isotype CBS-H 7633 not seen, ex-isotype culture CBS 439.82). *Phomopsis fukushii*, Japan, Ibaraki, on *Pyrus pyrifolia*, August 1994, S. Kanematsu, (neotype BPI 892933, ex-neotype culture MAFF625034 = AR3672).

Known distribution: Austria, China, France, Germany, Italy, Japan, Korea, Latvia, Lithuania, Netherlands, New Zealand, Poland, Portugal, Russia, Serbia, South Africa, UK, USA, Yugoslavia [54].

Host range: *Abutilon* sp., *Acer campestre*, *Acer nugundo*, *Alliaria officinalis*, *Allium giganteum*, *Arctium* sp., *Banksia* sp., *Betula alleghaniensis*, *Camelia sinensis*, *Castanea mollissima*, *C. sativa*, *Celastrus scandens*, *Chamaecyparis thyoides*, *Citrus* sp., *Cotoneaster* sp., *Cornus florida*, *Corylus avellena*, *Cucumis* sp., *Daphne lauriola*, *Eucalyptus globulus*, *Fraxinus excelsior*, *Glycine max*, *Hedera helix*, *Hordeum* sp., *Ilex aquifolium*, *Juglans cinerea*, *Juglans regia*, *Juniperus* sp., *Laburnum* sp., *Laurus nobilis*, *Magnolia* sp., *Malus pumila*, *M. silvestris*, *Opuntia* sp., *Osmanthus aquifolium*, *Oxydendrum arboreum*, *Phaseolus vulgaris*, *Picea pungens*, *P. glauca*, *P. abies*, *Pinus pantepylla*, *P. pinaster*, *Prunus persica*, *P. cerasus*, *P. nume*, *Pyrus pyrifolia*, *Quercus suber*, *Rhododendron* sp., *Rubus* sp., *Rubrus fructicosus*, *Rumex hydrolapathum*, *Salix* sp., *Sassafras albida*, *Skimmia japonica*, *Sorbus aucuparia*, *Tilis cordata*, *Ulmus minor*, *U. laevis*, *U. campestris*, *Vaccinium corymbosum*, *V. macrocarpon*, *Viburnum lantana*, *Vitis vinifera*, *Wisteria sinensis*, *Ziziphus jujuba* [54].

Description: Both sexual and asexual morphs of *D. eres* have been previously described and illustrated in detail by Udayanga et al. [23].

Notes: The micromorphology of the asexual morph of all species belonging to the *D. eres* complex match the original description of *D. eres* reported by Udayanga et al. [23]: pycnidia embedded in tissue, irregularly distributed over agar surface, producing abundant and black stromata at maturity, covered in white mycelium, and oozing yellowish conidial cirrhus from ostioles. Cultural characteristics are also similar to those reported by Udayanga et al. [23]: colonies spreading on PDA in a radial pattern with white, aerial, cottony mycelium, sometimes with brown aerial mycelium at the center, becoming grey at edges of plate, and reverse white to ivory color concentric zones, becoming brownish to black with age; conidiophores, alpha conidia and beta conidia with dimensions that correlate with the ones reported by those authors. Although the asexual morph of *D. neilliae* was not recorded by Udayanga et al. [23], the sexual morph was compared to the one of *D. eres*: perithecia and ascospores of *D. neilliae* that match within the same ranges as those of *D. eres*, and asci of *D. neilliae* were reported to be slightly shorter than those of *D. eres* (45 ± 5 × 8.5 ± 0.7 μm for *D. neilliae* vs. 53 ± 5 × 8.0 ± 0.7 for *D. eres*).

## 5. Discussion

In recent years, the use of multilocus DNA sequence data, coupled with morphology and ecology, has been widely employed to establish robust species boundaries in the genus *Diaporthe* [4,66]. Nevertheless, given that several authors have identified distinct species based on the concatenation of genes, without looking for incongruences between individual gene genealogies [9], the number of species in *Diaporthe* has been increasing considerably. This is largely attributed to the intraspecific variability in the genus, where each clade has been incorrectly recognized as a distinct lineage [21,67].

Although the ITS region is considered the primary barcode for fungi [68], it has been argued by several authors that this ribosomal DNA region harbors a high variability, and therefore it is believed to be uninformative to resolve species within the *D. eres* complex [69]. Considering this, the ITS region has been excluded from the phylogenetic analysis, and currently the concatenation of *TEF1-α*, *TUB2*, *HIS3* and *CAL* loci is widely used to resolve species in the aforementioned complex [16,18,21,23,24].

Despite Udayanga et al. [23] having proven that the concatenation of seven loci to resolve the *D. eres* complex, excluding the discordant ITS data, results in a robust tree congruent with the other single genes, the same was not verified in the present study. By applying the GCPSR principle, the genealogical concordance among genes must be verified [6]. However, incongruent nodes, conflicting branches, and the lack of phylogenetic support in some branches were observed in the individual phylograms versus the 4-loci and 5-loci combined trees. For example, isolates of *D. eres* (e.g., MAFF265034 and CFCC 51632, previously known as *D. fukushii* and *D. camptothecicola*, respectively) did not cluster together on the individual ITS tree but formed a monophyletic clade in the remaining individual and combined trees. Moreover, it was also evident that the species *D. brevicancria* was phylogenetically indistinguishable from *D. celastrina* on the individual *CAL* locus. Hence, by applying the genealogical concordance, we verified that the node delimiting the *D. eres* complex represents the transition from concordant branches to incongruence, thus indicating that this complex of species represents in fact one single species, *D. eres*. These incongruences observed between the individual gene genealogies confirm that the loci may harbor different evolutionary histories [70], thus making the concatenation of genes an inappropriate approach to infer the phylogenetic relationships within the *D. eres* complex.

Bearing in mind that the Ecological Species Concept recognizes species as of a group of individuals that occupies a specific niche [71], the use of phylogenetic approaches based on multiple loci aids in revealing ecological patterns of diversification among clades [7,72]. However, in the present study, we demonstrate that the species belonging to the *D. eres* complex are distributed worldwide, thus lacking a correlation between the genetic divergence of the complex and its ecological niche. Despite *D. pulla* being restricted to a specific locality (Yugoslavia), it was previously shown to belong to an unresolved sub-clade, which Gomes et al. [21] referred to as *D. nobilis* complex. Later, Udayanga et al. [23] showed that many of the isolates in the aforementioned complex clustered within *D. eres*, based on the GCPSR principle. Additionally, given the genetic differences between *D. pulla*, with its closest relative *D. helicis*, Udayanga et al. [23] suggested that these two species should be designated as distinct lineages. However, according to our results, *D. pulla* and *D. helicis* belong to *D. eres* complex and are thus synonymized here with *D. eres*.

Although the Morphological Species Concept was historically the dominant concept in the genus *Diaporthe* [19], the presence of conserved morphological characters made this concept inadequate to delineate species in this genus [20]. Based on the taxonomic description of the *D. eres* complex, a clear absence of morphological distinctiveness was evident. Overall, species harbor oblong to ellipsoid alpha conidia, a common presence of beta conidia, creamy to yellowish conidial cirrhus, conidiomata shapes and conidiophores dimensions that match within the same ranges. Though dendrograms of the length and width ratios distinguished the species into different groups, these are not correlated to any of the clades in the combined gene trees. Moreover, though there was higher variability in the L/W ratios observed for beta conidia, this might simply represent a character plasticity of *Diaporthe* rather than an indication of morphospecies. For instance, temperatures above 30 °C or the dextrose concentration (a component of the PDA medium) seems to influence the production of this type of conidia [20]. Culture characteristics are also quite identical among the recognized “species” within the *D. eres* complex: aerial mycelium cottony with yellowish gray to brownish-gray coloration, margin regular, producing abundant black stromata at maturity in culture and oozing yellow cirrhus. Therefore, we consider those micromorphological characters as minor differences among species, demonstrating only a high character variation within the *D. eres* complex. As stated by Hyde et al. [2], the recognition of significant recombination within closely related taxa should be considered as a method to justify a species. Therefore, the *D. eres* species complex was tested to disclose the presence of genetic recombination. Our results proved that significant genetic exchange occurs within the complex, indicating that there is no reproductive isolation between some of the putative species recognized on the 5-loci combined tree. The genetic diversity within the *D. eres* complex was also estimated. Low nucleotide diversity values in addition to high haplotype diversity indices indicate a high number of closely related haplotypes [73]. Moreover, although *TEF1-α* locus showed the highest informative characters to resolve the *D. eres* complex, as also corroborated by previous studies [23,25], the Tajima’s D test gave negative values for *TEF1-α* locus and the remaining genes (except for the ITS locus), which is indicative of inbreeding events within the population occurring at these loci. Therefore, this suggests that *D. eres* complex is a population that may have undergone a recent expansion, producing a large number of offspring [73,74]. In a recent study, Chaisiri et al. [25] also showed high levels of haplotype diversity within the five loci among *D. eres*, thus reflecting high genetic diversity. The neutrality Tajima’s test run by these authors similarly showed negative values, which suggests a population expansion in *D. eres* isolates. Such population expansion shown in the present study might be explained by inbreeding events among some species of the complex, occurring mainly in *TUB2* and ITS loci, showing significant genetic recombination. Therefore, taking into consideration the lack of gene flow, the absence of supporting phenotypic, geographic, and ecological differences, the recent divergence and the possibility of incomplete lineage sorting of the *D. eres* complex may be considered as ongoing evolving lineages [75].

Given the existence of conflicts between individual gene trees, the impossibility to combine genes and the lack of phenotypic distinctiveness, we attempted to delimit the species boundaries of the *D. eres* species complex through coalescent-based models. This latter approach involves understanding how several species are related by modeling the genealogical history of individuals with a common ancestor [7,13,76]. The General Mixed-Yule Coalescent (GMYC) and the Poisson Tree Processes (PTPs) models are widely used to identify branching patterns between divergence and intraspecific diversification and thus to distinguish between species and populations [77]. However, the GMYC model can overestimate the number of taxa, particularly in species with a strong intraspecific genetic structure [75]. An important advantage of using PTP analyses (both single and multi-rate) is that it models speciation events in terms of the number of nucleotide substitutions [13]. Therefore, it avoids the computation-intensive process of generating time-calibrated ultrametric trees, which are required as an input for GMYC analysis. Another drawback of this latter analysis is the choice of the molecular clock, which is essential to infer the timing of evolutionary divergence events in a given phylogeny [78]. However, this is somehow difficult to predict, as fossil evidence remains scarce for fungi [79]. For this reason, PTP analysis is considered to yield more accurate delimitations than GMYC [80], so this was the one adopted in the present study. According to our results, the highly supported clades recognized as distinct species in the combined 5-loci and 4-loci trees were not concordant with the coalescent methods, as both single and multi-rate PTP analyses inferred that *D. eres* complex should be recognized as a single species. Furthermore, based on the phylogenetic network performed, it is notable that the species within the *D. eres* complex are linked with boxes, which is indicative of the presence of recombination. For this reason, we considered that all species falling into the *D. eres* complex should be regarded as one single species, i.e., *D. eres*, rather than different taxa. According to Yang et al. [18], the species *D. maritima*, *D. phragmitis* and *D. rosicola* belong to the *D. eres* species complex. Given the lack of sequences available for some loci, these species could not be included in the analyses. Nevertheless, considering that these species fall into the *D. eres* complex, based in the individual genealogies performed, and knowing that Chasiri et al. [25] synonymized *D. rosicola* as *D. eres*, which we also corroborate in the present study, we feel comfortable considering *D. maritima* and *D. phragmitis* as synonymous with *D. eres*.

Of particular relevance is the synonymization of *D. vaccinii* with *D. eres*. *Diaporthe vaccinii* has been regarded as a common and important pathogen of the *Vaccinium* species, especially in the US, where it was originally reported [26]. In Europe, it is a quarantine organism and is regarded as eradicated from all countries where it was previously detected [81]. We recognize the potential impact that our proposal may have in the plant pathology community, but the results from the integrative approach performed in this study provide strong evidence that *D. vaccinii* cannot be regarded as a distinct species from *D. eres*. Previous studies recognize that both species are morphologically indistinguishable and phylogenetically very closely related [16,17,23,24,26]. Thus, it is not unreasonable to accept that they represent a single species.

The species *D. vaccinii* was described in an epoch where host association was regarded as an important character to delimit species. It is now widely accepted that most (if not all) species of *Diaporthe* are not host specific [19,20], and *D. vaccinii* is one of the rare exceptions still accepted. Recognizing it as a synonym of *D. eres* means that its status as a major pathogen of blueberry would need to be reassessed. Previous studies [82] have suggested that *D. vaccinii* is probably not a major threat to blueberry production in Europe and that its status as a quarantine organism should be reappraised.

Following the most common species concept used in fungi, the Phylogenetic Species Concept (PSC) [6], a species is assigned to a phylogenetic cluster that shares a most recent common ancestor, and it differs phenotypically from its closest relatives [8,10]. However, this is not always observed in the genus *Diaporthe*, as in the case of the *Diaporthe eres* complex, which was shown in this study to display little or no morphological variation. Moreover, the incongruences observed among the individual genealogies make the concatenation of multiple loci an inappropriate approach to delimit species. For this reason, we believe that the genealogical concordance allied to the recognition of significant recombination among species must be applied in future studies to delimit the species boundaries in the genus *Diaporthe*.

## 6. Conclusions

In the present study, phylogenetic analyses based on the GCPSR principle and the coalescent-based species model, PTP, prove that the *D. eres* complex is a population with evolving lineages, rather than a complex composed of distinct species. Furthermore, the pairwise homoplasy index test and the comparison of morphological and ecological characters highlight the absence of gene flow within the population, given that there is no evidence of reproductive isolation or of geographical barriers. This study suggests that the identification of species in *Diaporthe* has been largely overestimated, since the use of multilocus DNA sequences has been widely used without comparing the individual genealogies to look for incongruent nodes. This is particularly important in the genus *Diaporthe*, given the presence of a high intraspecific variability that might have been erroneously regarded as an aspect to describe novel taxa. Hence, individual gene genealogies must always be compared to look for incongruences among them. Once incongruent branches or a lack of phylogenetic support is observed, careful assumptions need to be made prior the description of new species in the genus *Diaporthe*. We also recommend that several strains from different locations should be included in the analyses, whenever possible, in the attempt to assess the intraspecific variation. Moreover, bearing in mind that the ITS region is the primary barcode for fungi, and it has been adopted as the genetic marker of choice for species delimitation, we advocate that this ribosomal region should not be excluded from the phylogenetic analyses, but carefully analyzed along with the other protein coding genes used in the genus *Diaporthe*, such as *TEF1-α*, *TUB2*, *HIS3* and *CAL* loci. In addition, further studies based on the coalescent models should also be implemented in the genus *Diaporthe* to provide stronger support to infer the phylogenetic relationships between cryptic species. Large-scale whole genome sequencing must also be considered in the future to provide insights into the validity of the current five loci used for molecular identification in the genus *Diaporthe*, as well as to identify new markers to be used in the delimitation of species in this genus or even to develop a genome-based taxonomy approach to delimit species in the genus.

## Figures and Tables

**Table 1:** List of *Diaporthe* species used in this study.

Species	Strain ^1^	Host	Country	GenBank Accession				
				ITS	*TEF1-α*	*TUB2*	*HIS3*	*CAL*
*D. ambigua*	**CBS 114015**	*Pyrus communis*	South Africa	KC343010	KC343736	KC343978	KC343494	KC343252
	CBS 123210	*Foeniculum vulgare*	Portugal	KC343012	KC343738	KC343980	KC343496	KC343254
*D. amygdali*	**CBS 126679**	*Prunus dulcis*	Portugal	KC343022	KC343748	KC343990	KC343506	KC343264
	CBS 115620	*Prunus persica*	USA	KC343020	KC343746	KC343988	KC343504	KC343262
*D. citri*	CBS 134239	*Citrus cinensis*	USA	KC357553	KC357522	KC357456	MF418280	KC357488
	**CBS 135422**	*Citrus* sp.	USA	KC843311	KC843071	KC843187	MF418281	KC843157
*D. eres*	CPC 29331	*Vitis vinifera*	France	MG281034	MG281555	MG281207	KC343631	KC343389
	**CBS 138594**	*Ulmus laevis*	Germany	KJ210529	KJ210550	KJ420799	KC343637	KC343395
	CBS 143344	*Vitis vinifera*	Czech Republic	MG281020	MG281541	MG281193	MG281366	MG281715
	CAA756	*Banksia* sp.	Portugal	*MW040531*	*MW052385*	*MW091320*	*MW052384*	*MW091319*
	CAA954	*Pinus pinaster*	Portugal	MN190309	MT309431	MT309457	MT309440	MT309448
	CAA1001	*Quercus suber*	Portugal	MT237172	MT309432	MT309458	MT309441	MT309449
*D. eres* (syn. *D. alnea*)	**CBS 146.46**	*Alnus* sp.	The Netherlands	KC343008	KC343734	KC343976	KC343492	KC343250
	CBS 159.47	*Alnus* sp.	The Netherlands	KC343009	KC343735	KC343977	KC343493	KC343251
*D. eres* (syn. *D. alleghaniensis*)	**CBS 495.72**	*Betula alleghaniensis*	Canada	FJ889444	GQ250298	KC843228	KC343491	KC343249
*D. eres* (syn. *D. betulae*)	**CFCC 50469**	*Betula platyphylla*	China	KT732950	KT733016	KT733020	KT732999	KT732997
	CFCC 50470	*Betula platyphylla*	China	KT732951	KT733017	KT733021	KT733000	KT732998
*D. eres* (syn. *D. betulina*)	**CFCC 52562**	*Betula platyphylla*	China	MH121497	MH121539	MH121579	MH121457	MH121421
	CFCC 52561	*Betula platyphylla*	China	MH121496	MH121538	MH121578	MH121456	MH121421
*D. eres* (syn. *D. bicincta*)	**CBS 121004**	*Juglans* sp.	USA	KC343134	KC343860	KC344102	KC343618	KC343376
*D. eres* (syn. *D. biguttusis*)	**CGMCC 3.17081**	*Lithocarpus glabra*	China	KF576282	KF576257	KF576307	-	-
*D. eres* (syn. *D. brevicancria*)	**CBS 146962**	*Picea pungens*	USA	MN136180	MN136153	MN136190	MN136178	MN136129
	MIFCC 305	*Picea glauca*	USA	MN136184	MN136151	MN136188	MN136176	MN136127
*D. eres* (syn. *Diaporthe* cf. *nobilis*)	**CBS 124030**	*Malus pumila*	New Zealand	KC343149	KC343875	KC344117	KC343633	KC343391
	CBS 134470	*Castanea sativa*	Australia	KC343146	KC343872	KC344114	KC343630	KC343388
	CBS 587.79	*Pinus pentaphylla*	Japan	KC343153	KC343879	KC344121	KC343637	KC343395
*D. eres* (syn. *D. camptothecicola*)	**CFCC 51632**	*Camptotheca acuminata*	China	KY203726	KY228887	KY228893	KY228881	KY228877
	CFCC 51633	*Camptotheca acuminata*	China	KY203727	KY228888	KY228894	KY228882	KY228878
*D. eres* (syn. *D. castaneae-mollissimae*)	**DNP128**	*Vaccinium corymbosum*	China	KC763096	KJ210561	KJ420801	KJ420852	KJ435040
*D. eres* (syn. *D. celeris*)	**CBS 143349**	*Vitis vinifera*	UK	MG281017	MG281538	MG281190	MG281363	MG281712
	CBS 143350	*Vitis vinifera*	UK	MG281018	MG281539	MG281191	MG281364	MG281713
*D. eres* (syn. *D. celastrina*)	**CBS 139.27**	*Celastrus* sp.	USA	KC343047	KC343773	KC344015	KC343531	KC343289
*D. eres* (syn. *D. chensiensis*)	**CFCC 52567**	*Abies chensiensis*	China	MH121502	MH121544	MH121584	MH121462	MH121426
	CFCC 52568	*Abies chensiensis*	China	MH121503	MH121545	MH121585	MH121463	MH121427
*D. eres* (syn. *D. cotoneastri*)	CBS 135428	*Juglans cinerea*	USA	KC843328	KC843121	KC843229	KC343630	KC343388
	**CBS 439.82**	*Cotoneaster* sp.	UK	KC343090	KC343816	KC344058	KC343574	KC343332
*D. eres* (syn. *D. ellipicola*)	**CGMCC 3.17084**	*Lithocarpus glabra*	China	KF576270	KF576245	KF576291	-	-
*D. eres* (syn. *D. fukushii*)	CBS 116953	*Pyrus pyrifolia*	New Zealand	KC343147	KC343873	KC344115	KC343631	KC343389
	**CBS 116954**	*Pyrus pyrifolia*	Japan	JQ807469	JQ807418	KJ420819	KJ420868	KJ435023
*D. eres* (syn. *D. helicis*)	**CBS 138596**	*Hedera helix*	France	KJ210538	KJ210559	KJ420828	KJ420875	KJ435043
*D. eres* (syn. *D. longicicola*)	**CGMCC 3.17089**	*Lithocarpus glabra*	China	KF576267	KF576242	KF576291	-	-
	CGMCC 3.17090	*Lithocarpus glabra*	China	KF576268	KF576243	KF576292	-	-
*D. eres* (syn. *D. mahothocarpus*)	**CGMCC 3.15181**	*Lithocarpus glabra*	China	KC153096	KC153087	KF576312	-	-
*D. eres* (syn. *D. maritima*)	**DAOMC 250563**	*Picea rubens*	Canada	KU552027	KU552022	KU574616	-	-
*D. eres* (syn. *D. momicola*)	**MFLUCC 16-0113**	*Prunus persica*	China	KU557563	KU557631	KU557587	-	KU557611
	MFLUCC 16-0114	*Prunus persica*	China	KU557564	KU557632	KU557588	-	KU557612
*D. eres* (syn. *D. neilliae*)	**CBS 144.27**	*Sapiraea* sp.	USA	KC343144	KC343870	KC344112	KC343628	KC343386
*D. eres* (syn. *D. padina*)	**CFCC 52590**	*Prunus padus*	China	MH121525	MH121567	MH121604	MH121483	MH121443
	CFCC 52591	*Malus domestica*	China	MH121526	MH121568	MH121605	MH121484	MH121444
*D. eres* (syn. *D. phragmitis*)	**CBS 138897**	*Phragmites australis*	China	KP004445	-	KP004507	KP004503	-
*D. eres* (syn. *D. pulla*)	**CBS 338.89**	*Hedera helix*	Yugoslavia	KC343152	KC343878	KC344120	KC343636	KC343394
*D. eres* (syn. *D. rosicola*)	MFLU 17-0646	*Rosa* sp.	UK	MG828895	MG829270	MG843877	-	MG829274
*D. eres* (syn. *D. vaccinii*)	**CBS 160.32**	*Vaccinium macrocarpon*	USA	AF317578	GQ250326	KC344196	KC343712	KC343470
	CBS 135436	*Vaccinium corymbosum*	USA	AF317570	JQ807380	KC843225	KJ420877	KC849457
*D. eres* (syn. *D. vacuae*)	CAA829	*Vaccinium corymbosum*	Portugal	MK792306	MK828077	MK837928	MK871446	MK883832
	**MUM 19.31**	*Vaccinium corymbosum*	Portugal	MK792309	MK828080	MK837931	MK871449	MK883834
*D. foeniculina*	CBS 123208	*Foeniculum vulgare*	Portugal	KC343104	KC343830	KC344072	KC343588	KC343346
	**CBS 111553**	*Foeniculum vulgare*	Spain	KC343101	KC343827	KC344069	KC343585	KC343343
*D. malorum*	CAA740	*Malus domestica*	Portugal	KY435642	KY435629	KY435670	KY435650	KY435660
	**CBS 142383**	*Malus domestica*	Portugal	KY435638	KY435627	KY435668	KY435648	KY435658
*D. sennicola*	**CFCC 51634**	*Senna bicapsularis*	China	KY203722	KY228883	KY228889	KY228879	KY228873
	CFCC 51635	*Senna bicapsularis*	China	KY203723	KY228883	KY228889	KY228879	KY228873

**^1^**Acronyms of culture collection: CAA—Personal Culture Collection Artur Alves, University of Aveiro, Aveiro, Portugal; CBS—Westerdijk Fungal Biodiversity Institute, Utrecht, The Netherlands; CFCC—China Forestry Culture Collection Center, Beijing, China; CGMCC—China General Microbiological Culture Collection Center, Beijing, China; CMW—Forestry and Agricultural Biotechnology Institute, University of Pretoria, South Africa; CPC—Personal Culture Collection, Pedro Crous, housed at CBS; DAOMC—The Canadian Collection of Fungal Cultures, Canada; DNP—Isolates in SMML culture collection, USDA-ARS, Beltsville, USA; MFLU—Herbarium of Mae Fah Luang University, Thailand; MIFCC—Michigan Isolate Fungal Culture Collection, Michigan, USA; MUM—Culture Collection from Micoteca da Universidade do Minho, Center for Biological Engineering of University of Minho, Braga, Portugal. Ex-type isolates are in bold. The new sequences generated in this study are in italics.

**Table 2:** Alignment properties and nucleotide substitution models used for phylogenetic analyses.

Character Status Summary	Loci and Combined Alignments						
	ITS	*TEF1-α*	*TUB2*	*HIS3*	*CAL*	4 loci	5 loci
Total characters	537	387	430	488	522	1827	2364
Invariable characters	434	183	273	342	307	1104	1539
Informative characters (%)	92 (17%)	184 (47%)	143 (33%)	116 (23%)	200 (38%)	649 (35%)	735 (31%)
Uninformative characters	11	20	14	30	15	74	90
Tree length (TL)	206	366	255	280	356	1358	1647
Consistency index (CI)	0.6359	0.7486	0.7686	0.7036	0.8090	0.7018	0.6594
Homoplasy index (HI)	0.3641	0.2514	0.2314	0.2964	0.1910	0.2982	0.3406
Retention index (RI)	0.8727	0.8892	0.8959	0.8683	0.9037	0.8554	0.8311
Rescaled consistency index (RC)	0.5549	0.6657	0.6886	0.6109	0.7311	0.6003	0.5480
Nucleotide substitution models ^1^	K2 + G	HKY	K2 + G	KKY + G	T92 + G	TN93 + G	TN93 + G

^1^ K2: Kimura 2-parameter model; T92: Tamura 3-parameter model; HKY: Hasegawa–Kishono–Yano model; TN93: Tamura-Nei parameter model; G: models of evolution assuming a gamma distribution.

**Table 3:** Polymorphism and genetic diversity data for the *Diaporthe eres* species complex.

Loci	Number of Haplotypes (h)	Polymorphic Sites (S)	Haplotype Diversity (hd)	Nucleotide Diversity (π)	Tajima’s D Test
ITS	18	35	0.948	0.025	0.6097
*TEF1-α*	19	43	0.966	0.019	−1.6281
*TUB2*	17	43	0.943	0.022	−0.7208
*HIS3*	13	44	0.882	0.021	−1.0077
*CAL*	12	13	0.921	0.009	−0.3513
5 loci ^1^	25	178	0.990	0.019	−0.7359
4 loci ^2^	24	143	0.988	0.018	−1.0881

^1^ Combined alignment based on ITS, *TEF1-α*, *TUB*, *HIS3* and *CAL* loci. ^2^ Combined alignments based on *TEF1-α*, *TUB2*, *HIS3* and *CAL* loci. Note: all Tajima’s D tests resulted in values that were not statistically significant.

**Table 4:** Synopsis of morphological characteristics of *Diaporthe eres* synonymous discussed in this study. (Note: the taxonomic description of *D. neilliae* was not included as its asexual morph is unknown).

Species	Conidiomata	Conidiophores and Conidiogenous Cells	Alpha Conidia (µm)	Beta Conidia (µm)	References
*D. alleghaniensis* Arnold 1967 *	Conidiomata small, with a conical shape, 200–250 µm diameter	Conidiophores 9–15 × 1–2 μm, hyaline, unbranched, ampulliform, cylindrical to sub-cylindrical. Conidiogenous phiailidic, cylindrical, slightly tapering towards apex.	Alpha conidia 7–9 × 3–4 μm, aseptate, hyaline, smooth, ovate to ellipsoidal, biguttulate or multiguttulate, base sub-truncate.	Not observed	[23]
*D. alnea* Fuckel 1870 * =*Phomopsis alnea*	Pycnidia with 100–200 μm diameter, globose to subglobose, embedded in tissue, erumpent at maturity	Conidiophores 9–16 × 1–2 μm, hyaline, unbranched, ampulliform, cylindrical to sub-cylindrical. Conidiogenous cells phiailidic, cylindrical, tapering towards the apex.	Alpha conidia 8–10 × 2–3 μm, aseptate, hyaline, smooth, ellipsoidal, biguttulate or multiguttulate, base subtruncate.	Not observed	[23]
*D. betulae* Tian and Fan 2016 *	Conidiomatal stromata immersed, erumpent, separate, conical, with a single locule	Conidiophores reduced to phiailidic conidiogenous cells hyaline, straight or slightly curved.	Alpha conidia hyaline, ellipsoidal, aseptate, smooth, biguttulate, 8.5–11.5 × 3.5–4.5 μm.	Not observed	[55]
*D. betulina* Tian and Yang 2018 *	Conidiomata pycnidial, conical, immersed and erumpent through the bark surface, 290–645 μm diameter	Conidiophores 12.5–17.5 × 1.5–2 μm, cylindrical, hyaline, phiailidic, branched, straight or slightly curved.	Alpha conidia hyaline, aseptate, ellipsoidal to fusiform, biguttulate, acute at both ends, 8–10 × 2.5–3 μm.	Beta conidia hyaline, aseptate, filiform, straight, eguttulate, tapering towards one apex, 26–32.5 × 1 µm	[18]
*D. bicincta* Cooke and Peck 1882 *	Pycnidia with 200–300 μm diameter, globose, erumpent at maturity, conidial cirrhus extruding from ostiole	Conidiophores 7–12 × 1–2 μm, hyaline, smooth, unbranched, cylindrical to sub-cylindrical. Conidiogenous cells 0.5–1 μm diameter, phiailidic, cylindrical, tapering towards the apex.	Alpha conidia 9–12 × 2–3.5 μm, aseptate, hyaline, ovate to ellipsoidal, biguttulate or multiguttulate, base subtruncate.	Not observed	[23]
*D. biguttusis* Gao and Cai 2015 *	Pycnidial conidiomata, dark brown, globose clustered, 79–227 μm diameter	Conidiophores 11.5–27.1 × 1.4–2.3 μm, cylindrical, single to multi-septate, densely aggregated, slightly tapering towards the apex.	Alpha conidia hyaline, biguttulate, fusiform or oval, with both ends obtuse, 5.9–8.5 × 1.9–2.6 μm.	Beta conidia hyaline, aseptate, filiform, hamate, eguttulate, tapering towards both ends, 28.1–37.9 × 1.3–2.0 μm	[56]
*D. brevicancria* Sakalidis and Medina-Mora 2021 *	Pycnidia dark brown to black, emersed in host tissue, solitary or aggregated, often with creamy yellow conidial cirrhus, 236–368 μm diameter	Conidiophores hyaline, reduced to conidiogenous cells phiailidic, and narrowing towards the apex, 7.1–17.5 × 1.0–2.4 μm.	Alpha conidia, hyaline, aseptate, oblong to ellipsoid, often biguttulate. with a sub-truncated base, 4.4–8.6 × 1.3–3.3 µm.	Beta conidia aseptate, hyaline, smooth, mostly convex at one end, hooked, 12.4–27.4 × 0.9–2.1 µm	[57]
*D. camptothecicola* Tiang and Yang 2017	Conidiomatal pycnidia immersed or slightly erumpent through bark surface, sparse, globose to ovoid, with 560 μm diameter	Conidiophores (8.3–)12.5–15.8(−17.0) × 0.9–1.2 μm hyaline, unbranched, smooth, cylindrical, straight or slightly curved, conidiogenous cells enteroblastic, phiailidic.	Alpha conidia hyaline, aseptate, oblong, biguttulate, (4.6–)5.5–7.0(−7.5) × 1.5–1.8 μm.	Beta conidia hyaline, aseptate, filiform with obtuse ends, 19.5–28.3 × 1.0 μm	[58]
*D. celastrina* Ellis and Barthol 1902 *	Pycnidia with 200–300 μm diameter, globose, embedded in tissue, erumpent at maturity conidial cirrhus extruding from ostiole	Conidiophores 7–21 × 1–2 μm, hyaline, smooth, unbranched, ampulliform, cylindrical. Conidiogenous cells 0.5–1 μm diam, phiailidic, cylindrical, terminal, slightly tapering towards apex.	Alpha conidia 9–12 × 2–3.5 μm, aseptate, hyaline, ellipsoidal, biguttulate, multiguttulate, or eguttulate, base subtruncate.	Not observed	[23]
*D. celeris* Guarnaccia, Woodhall and Crous 2018 *	Conidiomata pycnidial, globose or irregular, solitary, erumpent, dark brown to black, 350–650 μm diameter, with yellowish translucent to brown conidial cirrhus	Conidiophores hyaline, smooth, unbranched, cylindrical, straight, 5–18 × 1–3 μm. Conidiogenous cells phiailidic, hyaline, cylindrical, 5–8 × 1–2 μm, tapering towards the apex.	Alpha conidia, aseptate, fusiform, hyaline, mono- to biguttulate and acutely rounded at both ends, 5.5–7.5 × 2–3 μm.	Beta conidia hyaline, eguttulate, filiform, curved, tapering towards both ends, 16–22.5 × 1–2 μm	[17]
*D. chensiensis* Tian and Yang 2018 *	Conidiomata pycnidial, immersed in bark, slightly erumpent discoid, ostiolate, 200–325 μm diameter	Conidiophores 8.5–13 × 2–3 μm, cylindrical, hyaline, phiailidic, unbranched, straight or slightly curved, tapering towards the apex.	Alpha conidia hyaline, aseptate, smooth, ellipsoidal, biguttulate, rounded at both ends, 6.5–11 × 2–2.2 μm.	Beta conidia present on the host only, hyaline, eguttulate, smooth, filiform, 21–28.5 × 0.8–1.1 μm	[18]
*D. ellipicola* Gao and Cai 2015	Pycnidial conidiomata, globose, 141–338 µm diameter, erumpent, single or clustered, extruding yellowish translucent conidial droplets from the ostioles	Conidiophores cylindrical, branched, septate, hyaline, 12–22.4 × 1.1–2 µm, phiailidic, cylindrical, straight, slightly tapering towards the apex.	Alpha conidia 6–8.7 × 2–3 µm, aseptate, hyaline, smooth, biguttulate, oval, ellipsoid rounded at both ends.	Beta conidia 23.4–35.5 × 1.4–2 µm, hyaline, curved	[56]
*D. eres* Nitschke 1870 =*D. castaneae-mollisimae* ***=****D. cotoneastri* =*D. fukushi*	Pycnidia with 200–250 μm diameter, globose, embedded in tissue, erumpent at maturity, often with yellowish, conidial cirrhus extruding from ostiole	Conidiophores 10–15 × 2–3 μm, hyaline, smooth, unbranched, ampulliform, straight to sinuous. Conidiogenous cells 0.5–1 μm diameter, phiailidic, cylindrical, slightly tapering towards the apex.	Alpha conidia (6–)6.5–8.5(−9) × 3–4 μm, aseptate, hyaline, smooth, ovate to ellipsoidal, often biguttulate, base subtruncate.	Beta conidia 18–29 × 1–1.5 μm, aseptate, hyaline, smooth, fusiform to hooked, base sub-truncate	[23]
*D. helicis* Niessl 1876 * =*D. nitschkei*	Pycnidia with 200–300 μm diameter, globose, embedded in tissue, erumpent at maturity, often with white conidial cirrhus extruding from ostiole	Conidiophores (6–)8–15 (16.5) × 1–2 μm, hyaline, smooth, unbranched, ampulliform, cylindrical to clavate. Conidiogenous cells phiailidic, cylindrical, tapering towards the apex.	Alpha conidia (5.5–)6–8(9.5) × 2.5–3.5 μm, aseptate, hyaline, smooth, cylindrical to ellipsoidal, biguttulate or multiguttulate, base subtruncate.	Not observed	[23]
*D. longicicola* Gao and Cai 2015	Conidiomata pycnidial, globose to subglobose, 500–750 µm diameter, dark brown to black, covered with white mycelium	Conidiophores 14.1–22.5 × 1.3–2 µm, hyaline, branched, densely aggregated, cylindrical, tapering towards the apex.	Alpha conidia 5.3–10.4 × 1.5–3.1 µm, with two big guttulate or 2–3 small guttulate, hyaline, ellipsoid or clavate, with one end obtuse and the other end acute and elongate.	Beta conidia filiform, hyaline, hamate or curved, aseptate, 25–32.2 × 1.2–2 µm	[56]
*D. mahothocarpus* Gao and Cai 2015 =*Phomopsis mahothocarpus*	Conidiomata globose, 200–350 μm diameter, ostiolate, deeply embedded in culture, aggregated in clusters	Conidiophores 15.5–21.8 × 1.6–2.2 μm, cylindrical, hyaline, branched, septate, straight or slightly curved.	Alpha conidia 5.5–8.0 × 1.8–2.9 μm, hyaline, aseptate, oval or fusiform, usually with one guttule at each end.	Beta conidia 21.1–28.5 × 1.2–1.9 μm, aseptate, filiform, hyaline, curved, eguttulate, with obtuse ends	[59]
*D. maritima* Tanney 2016 *	Conidiomata pycnidial, globose to subglobose, unilocular/multilocular, aggregated, dark brown to black, ostiolate, up to 300 μm diameter, with yellowish conidial mass	Conidiogenous cells phiailidic, subcylindrical to ampulliform, straight to sinuous, cylindrical or slightly tapering towards the apex, (8.5–)9–12.5(–16) × 2–3 μm.	Alpha conidia aseptate, hyaline, smooth, oblong to fusiform or ellipsoidal, apex rounded, base subtruncate, bi- to multiguttulate (10–)11–12.5(–13.5) × (3–)3.5–4 μm.	Beta conidia aseptate, hyaline, smooth, straight to hamiform or uncinate 29–40 × 1–2 μm	[60]
*D. momicola* Dissanayake, Li and Hyde 2017	Conidiomata up to 350 μm diameter, solitary or in groups with black cylindrical ostiolate necks, subglobose	Conidiophores reduced to conidiogenous cells.	Alpha conidia 6.5–9.5 × 1.5–2 μm, hyaline, smooth, biguttulate, fusiform to oval, tapered at both ends, cylindrical to ellipsoidal.	Beta conidia 20–32 × 1–1.5 μm, scattered among the alpha conidia	[61]
*D. nobilis* Saccardo and Spegazzini 1878	Conidiomata pycnidial, scattered to confluent, uniloculate, dark brown to black, broadly spherical to flattened, 650–700 μm high and 400–500 μm wide	Conidiophores thin walled, brown, vertically aligned, multicellular, 2–6 μm wide, elongate. Conidiogenous cells formed at the apex of the conidiophores cylindric, straight or curved.	Alpha conidia 7–9 × 3–5 μm, aseptate, cylindrical or ellipsoidal, obtuse at both ends, hyaline, generally biguttulate.	Beta conidia 20–30 × 0.3–0.8 μm, filiform, blunt at one end, pointed and usually curved at the other, hyaline, one-celled	[62]
*D. padina* Tian and Yang 2017 *	Conidiomata pycnidial, immersed in bark, scattered, slightly erumpent, light brown, one ostiole, 330–520 μm diameter	Conidiophores 5.5–12.5 × 1–1.5 μm, hyaline, unbranched, cylindrical, straight or slightly curved.	Alpha conidia hyaline, aseptate, ellipsoidal to fusiform, eguttulate, 7–8 × 1.5–2 μm.	Beta conidia hyaline, filiform, straight or hamate, eguttulate, base truncate, 21–24 × 1 µm	[18]
*D. phragmitis* Crous 2014 *	Conidiomata pycnidial, globose, up to 250 µm diameter, black, erumpent, exuding creamy conidial droplets from central ostioles	Conidiophores hyaline, smooth, septate, rarely branched, densely aggregated, cylindrical, 20–30 × 3–4 µm. Conidiogenous cells phiailidic, cylindrical, terminal, intercalary.	Alpha conidia aseptate, hyaline, smooth, multi- or bi-guttulate, fusoid to ellipsoid, tapering towards both ends, base subtruncate, 6–8.5 × 2–3 µm.	Not observed	[63]
*D. pulla* Nitschke 1870 * =*Phoma pulla* =*Phomopsis pulla*	Pycnidia with 200–300 μm diameter, globose, embedded in tissue, erumpent at maturity, black stromata, with bright yellow conidial cirrhus	Conidiophores 10–25 × 1–2 μm, hyaline, unbranched, cylindrical to clavate. Conidiogenous cells phiailidic, cylindrical, slightly tapering towards the apex.	Alpha conidia (6–)6.5–7.5 (8) × (2–) 2.5–3.5(−4) μm, aseptate, hyaline, smooth, cylindrical to ellipsoidal, biguttulate or multi-guttulate, base subtruncate.	Not observed	[23]
*D. rosicola* Wanasinghe, Jones and Hyde 2018 *	Conidiomata pycnidial, 120–160 µm diameter, solitary, semi-immersed, unilocular, globose, dark brown, ostiolate	Conidiophores hyaline, smooth, unbranched, cylindrical, straight to sinuous. Conidiogenous cells phiailidic, cylindrical, slightly tapering towards the apex.	Alpha conidia 7–9.5 × 2.4–3 µm, hyaline, biguttulate, fusiform or oval, both ends, obtuse.	Beta conidia 12–22 × 1.2–1.6 µm, hyaline, aseptate, filiform, tapering towards both ends	[64]
*D. vaccinii* Shear 1931 * =*Phomopsis vaccinii*	Conidiomata superficial, scattered, black, spherical to irregular, uniloculate, with ostiole circular, exuding white to yellowish cirrhus	Conidiogenous cells enteroblastic, phiailidic, with conidiophores short, 1–2 septa or multiseptate, branched.	Alpha conidia 5.9–11.3 × 2.1–3.9 μm, hyaline, fusiform, straight, guttulate, aseptate.	Beta conidia hyaline, filiform, straight or curved, eguttulate, aseptate	[65]
*D. vacuae* Hilário, Santos and Alves 2020 *	Pycnidial conidiomata, brown to black, broadly spherical, covered in white mycelium, with yellowish conidial cirrhus extruding from ostiole	Conidiophores reduced to conidiogenous cells, hyaline, smooth and straight to sinuous, broadening in the base, slightly tapering toward the apex (10.9 ± 2.2 × 1.8 ± 0.3) μm.	Alpha conidia infrequent, hyaline, smooth, cylindrical, 9.3 ± 1.1 × 2.6 ± 0.3 μm.	Beta conidia hyaline, 1-celled, smooth, filiform, frequently hooked in apical part, apex acute, 27.4 ± 2.3 × 1.6 ± 0.2 μm	[29]

The newly synonymous introduced in the present study are marked with an asterisk (*).

## Data Availability

All data generated or analyzed in this study are included in this article and its supplementary information files. All sequence data are available in the NCBI GenBank, following the accession numbers in the manuscript.

## Supplementary Materials

The following are available online at https://www.mdpi.com/article/10.3390/jof7070507/s1, Figures S1–S5. Maximum Likelihood trees based on ITS, *TEF1-α*, *TUB2*, *HIS3* and *CAL* sequence data, respectively, for all species of the *Diaporthe eres* species complex. Figures S6–S10. Maximum Likelihood tree based on ITS, *TEF1-α*, *TUB2*, *HIS3* and *CAL* sequence data, respectively, for the *Diaporthe eres* species complex and related species. Figure S11. Maximum Likelihood tree based on *TEF1-α*, *TUB2*, *HIS3* and *CAL* sequence data for the *Diaporthe eres* species complex and related species. Figure S12. Results of the single PTP analyses for the *Diaporthe eres* species complex and related taxa, based on *TEF1-α*, *TUB2*, *HIS3* and *CAL* loci on Bayesian and Maximum Likelihood topologies.
