# Supplementary material for: Using Genealogical Concordance and Coalescent-Based Species Delimitation to Assess Species Boundaries in the Diaporthe eres Complex
Source: J Fungi (Basel). 2021 Jun 25;7(7):507. doi: 10.3390/jof7070507 (PMC8307253; doi:10.3390/jof7070507)
Supplement: Supplementary file 1 [file jof-07-00507-s001.zip › jof-1252647-SI.pdf]

Journal of Fungi

Supplementary Material

Using Genealogical Concordance and Coalescent-Based Species Delimitation to Assess Species Boundaries in the *Diaporthe eres* Complex

Sandra Hilário, Micael F. M. Gonçalves and Artur Alves *

Centre for Environmental and Marine Studies (CESAM), Department of Biology, University of Aveiro, Campus Universitário de Santiago, 3810-193 Aveiro, Portugal; sandra.hilario@ua.pt (S.H.); mfmg@ua.pt (M.F.M.G.)
* Correspondence: artur.alves@ua.pt

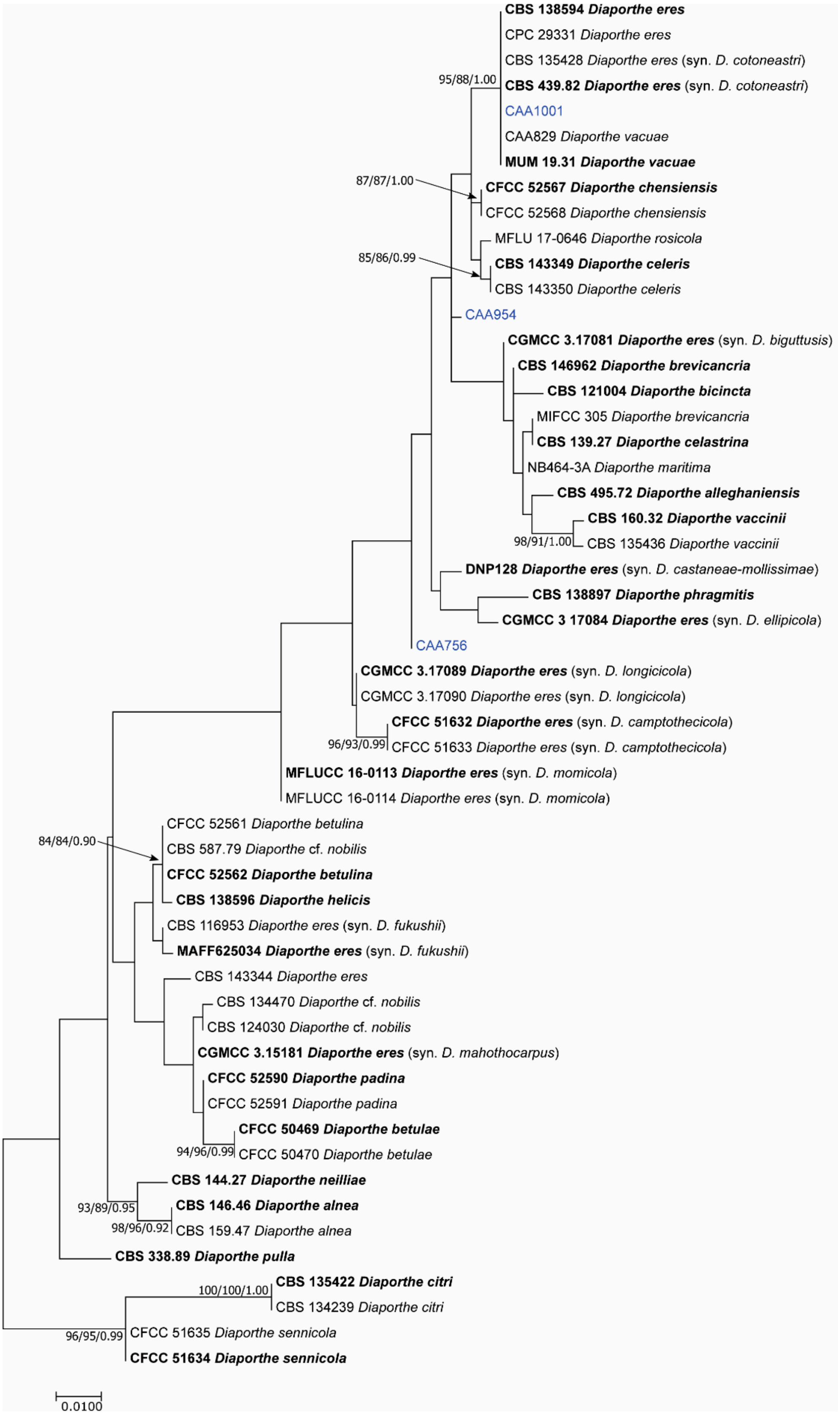

Figure S1. Maximum Likelihood trees based on ITS sequence data for all species of the Diaporthe eres species complex. The ML tree was constructed based on the Kimura 2-parameter model assuming a gamma distribution. Maximum Likelihood and Maximum Parsimony bootstrap values greater than 70% and posterior probabilities (PPs) inferred by Bayesian analysis greater than 0.80 are shown at the nodes. The ex-type strains are in bold. The newly generated sequences are indicated in blue.

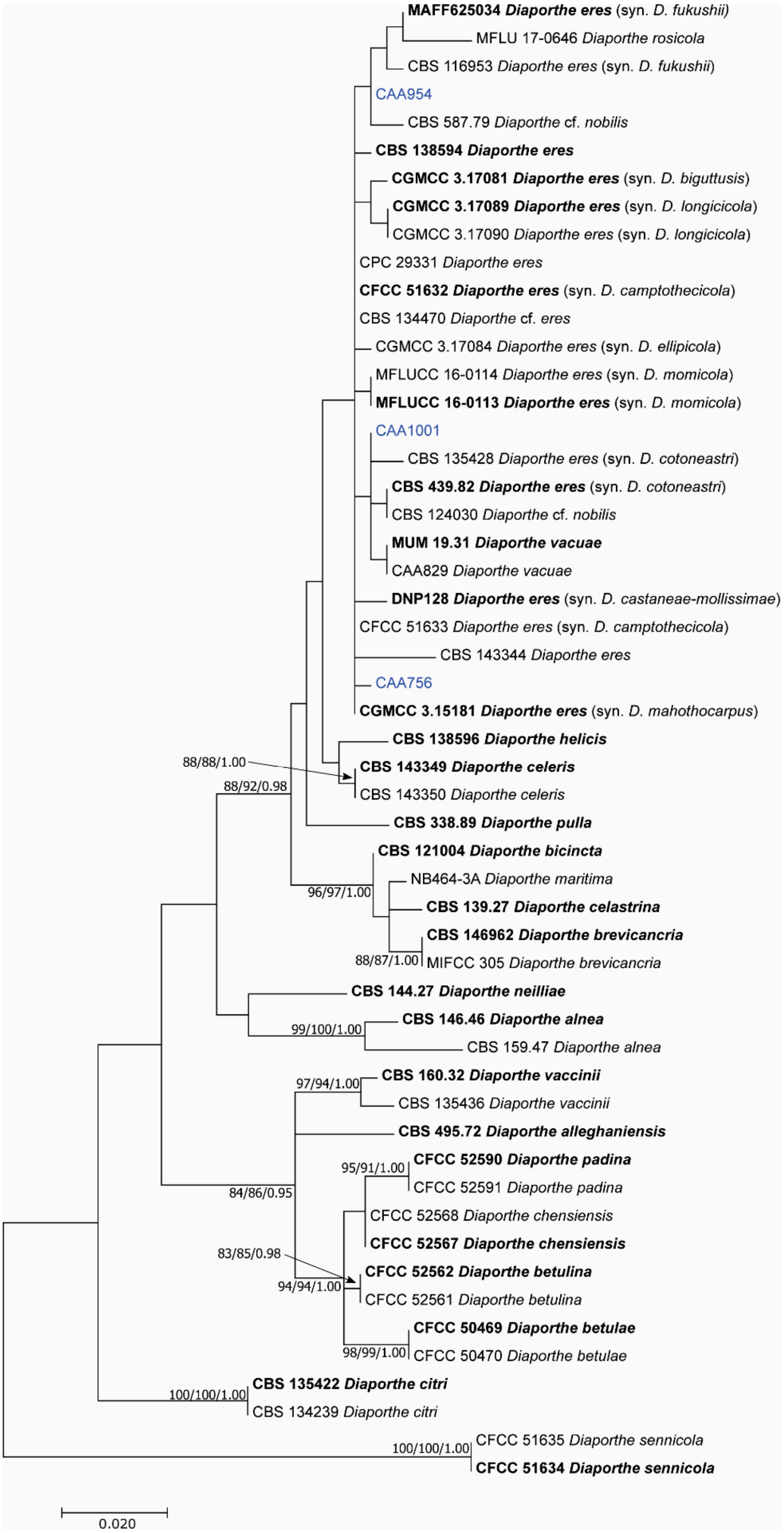

Figure S2. Maximum Likelihood trees based on TEF1-α sequence data for all species of the Diaporthe eres species complex. The ML tree was constructed based on the Kimura 2-paramter model assuming uniform rates. Maximum Likelihood and Maximum Parsimony bootstrap values greater than 70% and posterior probabilities (PPs) inferred by Bayesian analysis greater than 0.80 are shown at the nodes. The ex-type strains are in bold. The newly generated sequences are indicated in blue.

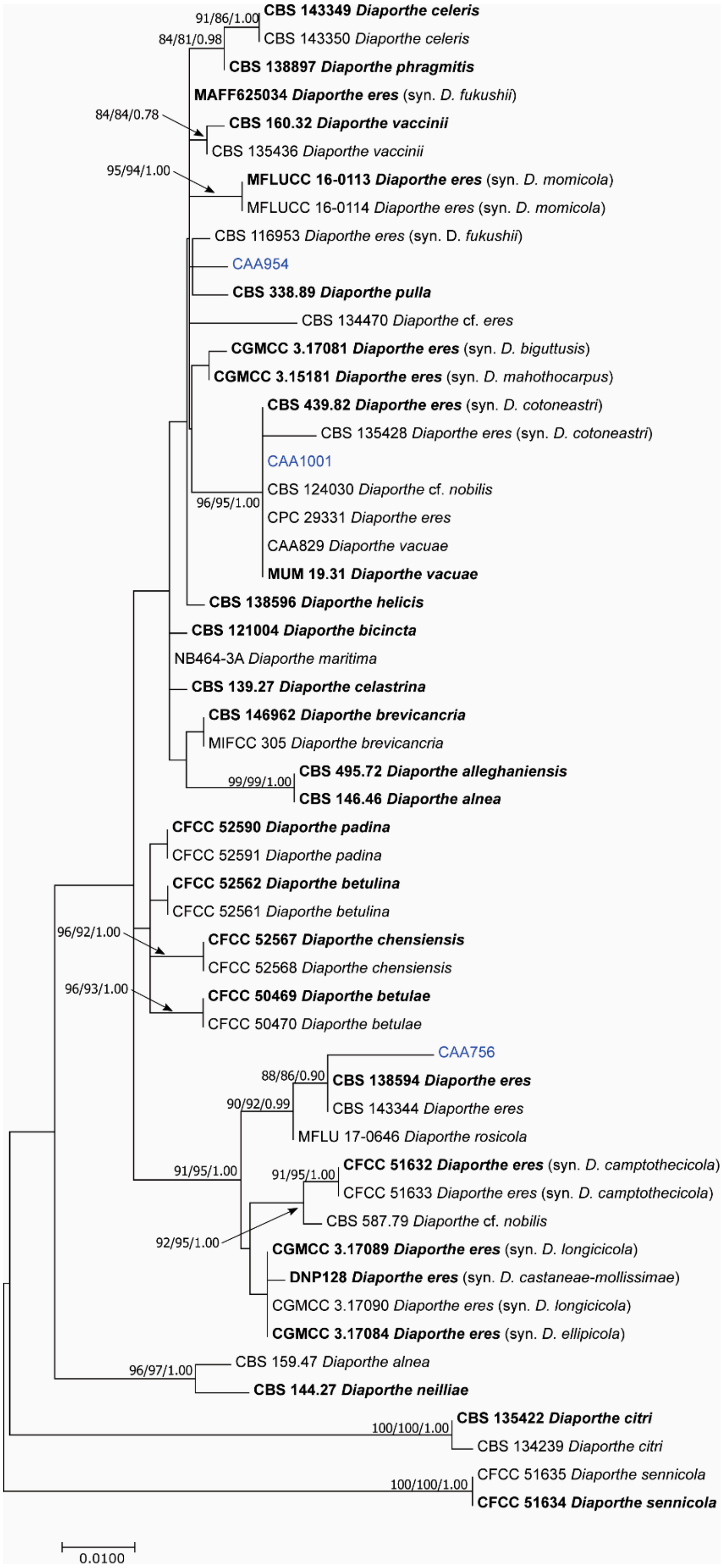

Figure S3. Maximum Likelihood trees based on TUB sequence data for all species of the Diaporthe eres species complex. The ML tree was constructed based on the Kimura 2-paramter model assuming a gamma distribution. Maximum Likelihood and Maximum Parsimony bootstrap values greater than 70% and posterior probabilities (PPs) inferred by Bayesian analysis greater than 0.80 are shown at the nodes. The ex-type strains are in bold. The newly generated sequences are indicated in blue.

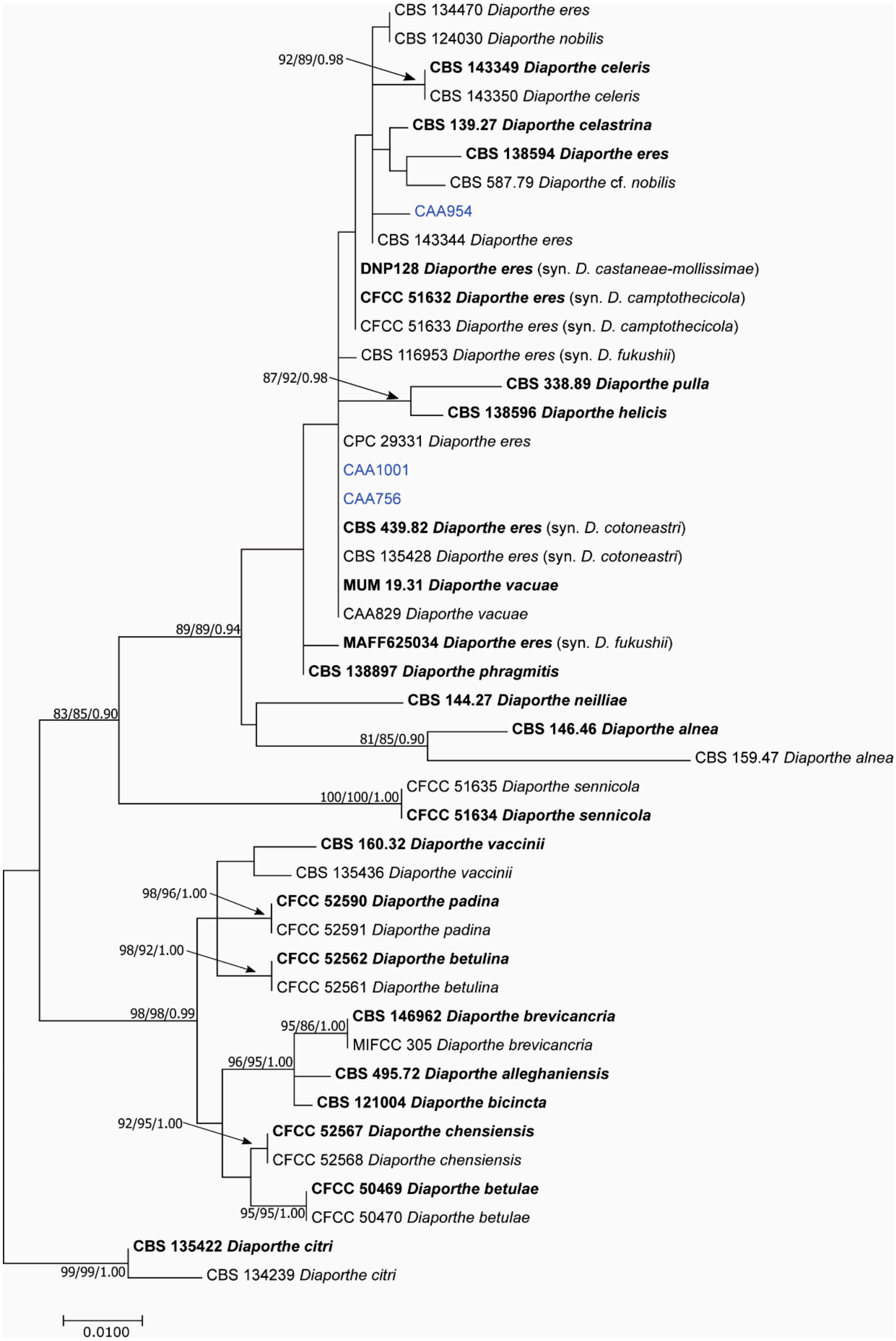

Figure S4. Maximum Likelihood trees based on HIS sequence data for all species of the Diaporthe eres species complex. The ML tree was constructed based on the Hasegawa-Kishino-Yano model assuming a gamma distribution. Maximum Likelihood and Maximum Parsimony bootstrap values greater than 70% and posterior probabilities (PPs) inferred by Bayesian analysis greater than 0.80 are shown at the nodes. The ex-type strains are in bold. The newly generated sequences are indicated in blue.

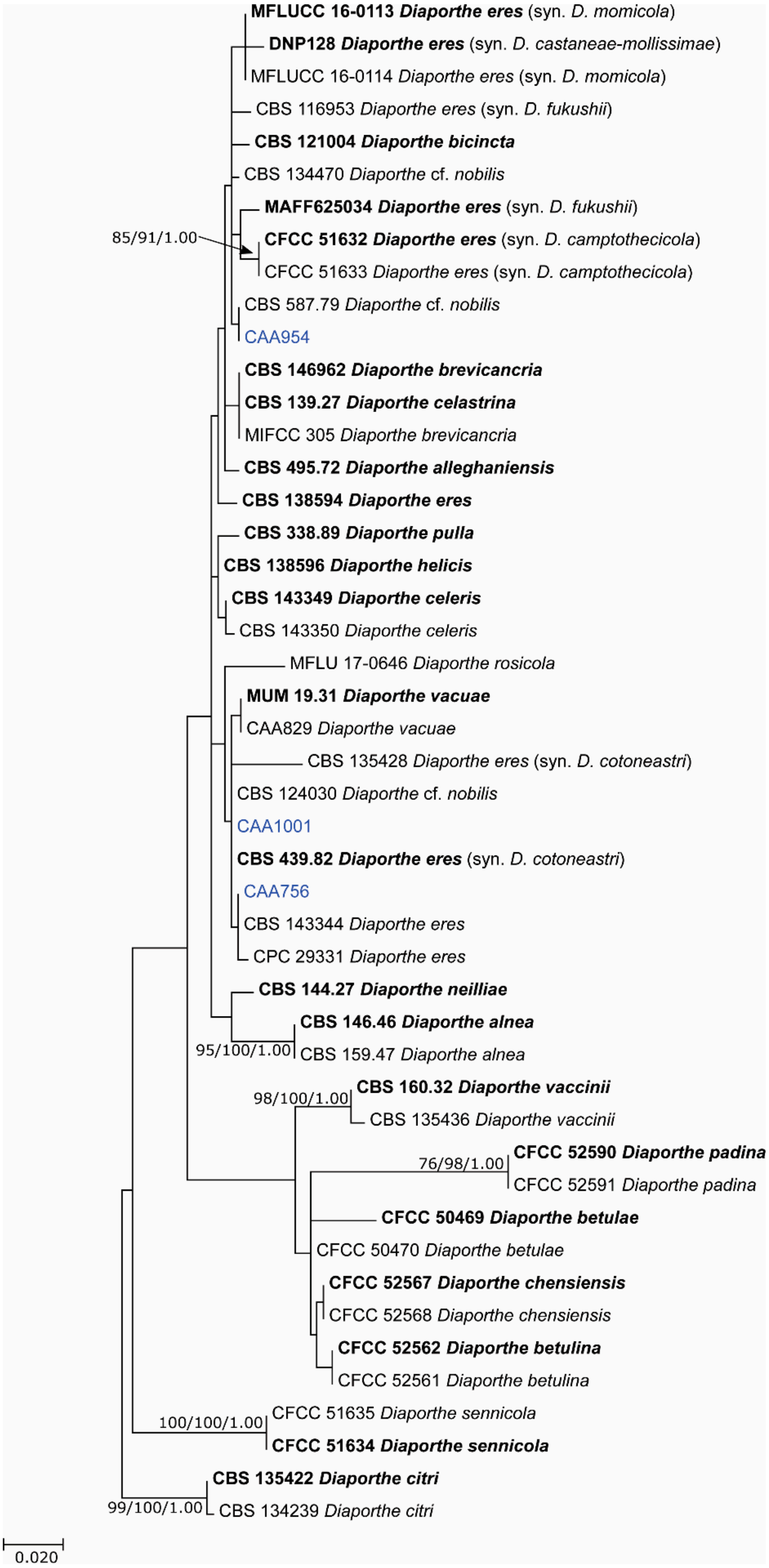

Figure S5. Maximum Likelihood trees based on CAL sequence data for all species of the Diaporthe eres species complex. The ML tree was constructed based on the Tamura 3-paramter model assuming a gamma distribution. Maximum Likelihood and Maximum Parsimony bootstrap values greater than 70% and posterior probabilities (PPs) inferred by Bayesian analysis greater than 0.80 are shown at the nodes. The ex-type strains are in bold. The newly generated sequences are indicated in blue.

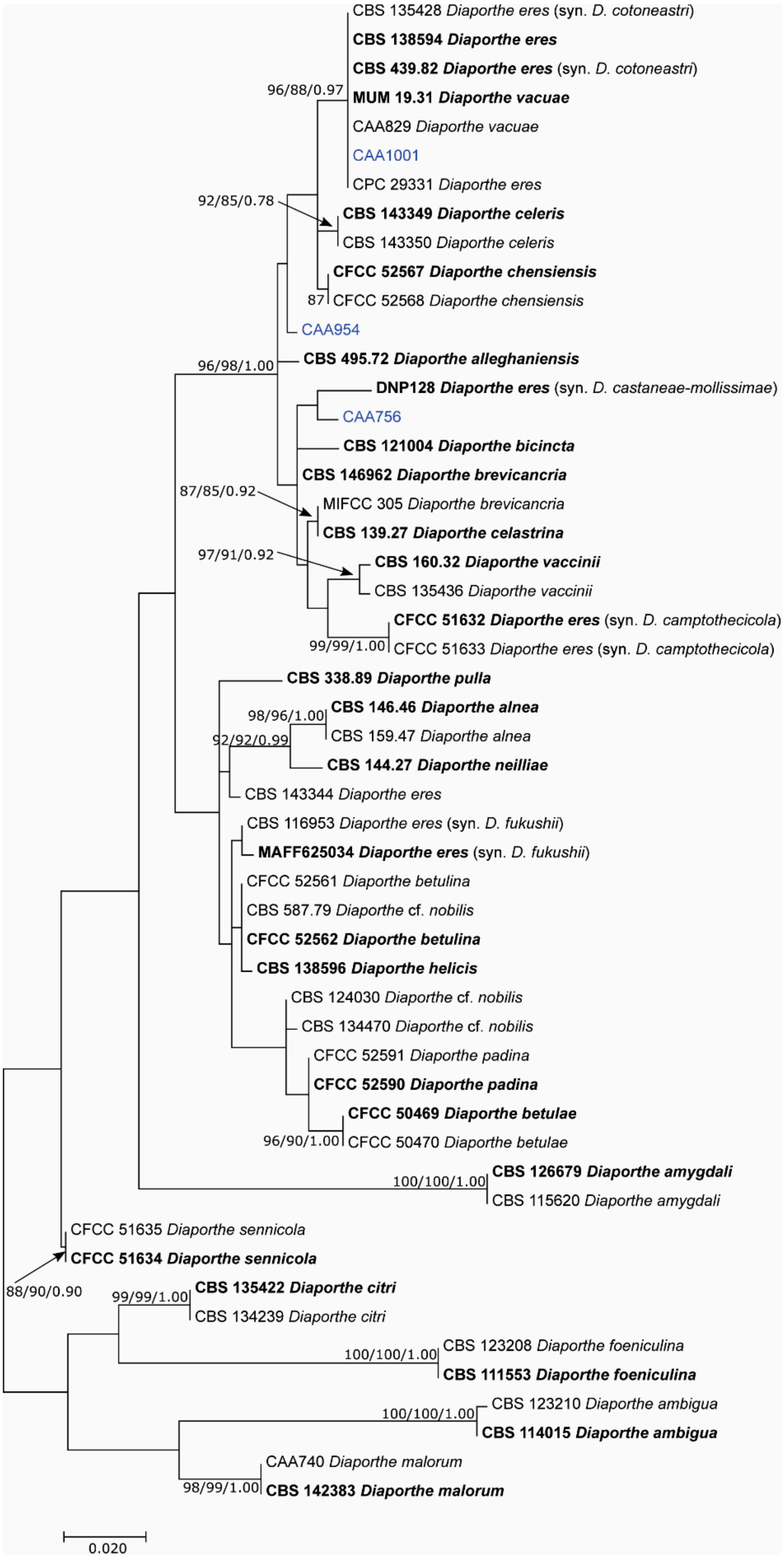

Figure S6. Maximum Likelihood tree based on ITS sequence data for the Diaporthe eres species complex and related species. The ML tree was constructed based on the Kimura 2-paramter model assuming a gamma distribution and invariant sites. Maximum Likelihood and Maximum Parsimony bootstrap values greater than 70% and posterior probabilities (PPs) inferred by Bayesian analysis greater than 0.70 are shown at the nodes. The ex-type strains are in bold. The isolates from this study are indicated in blue.

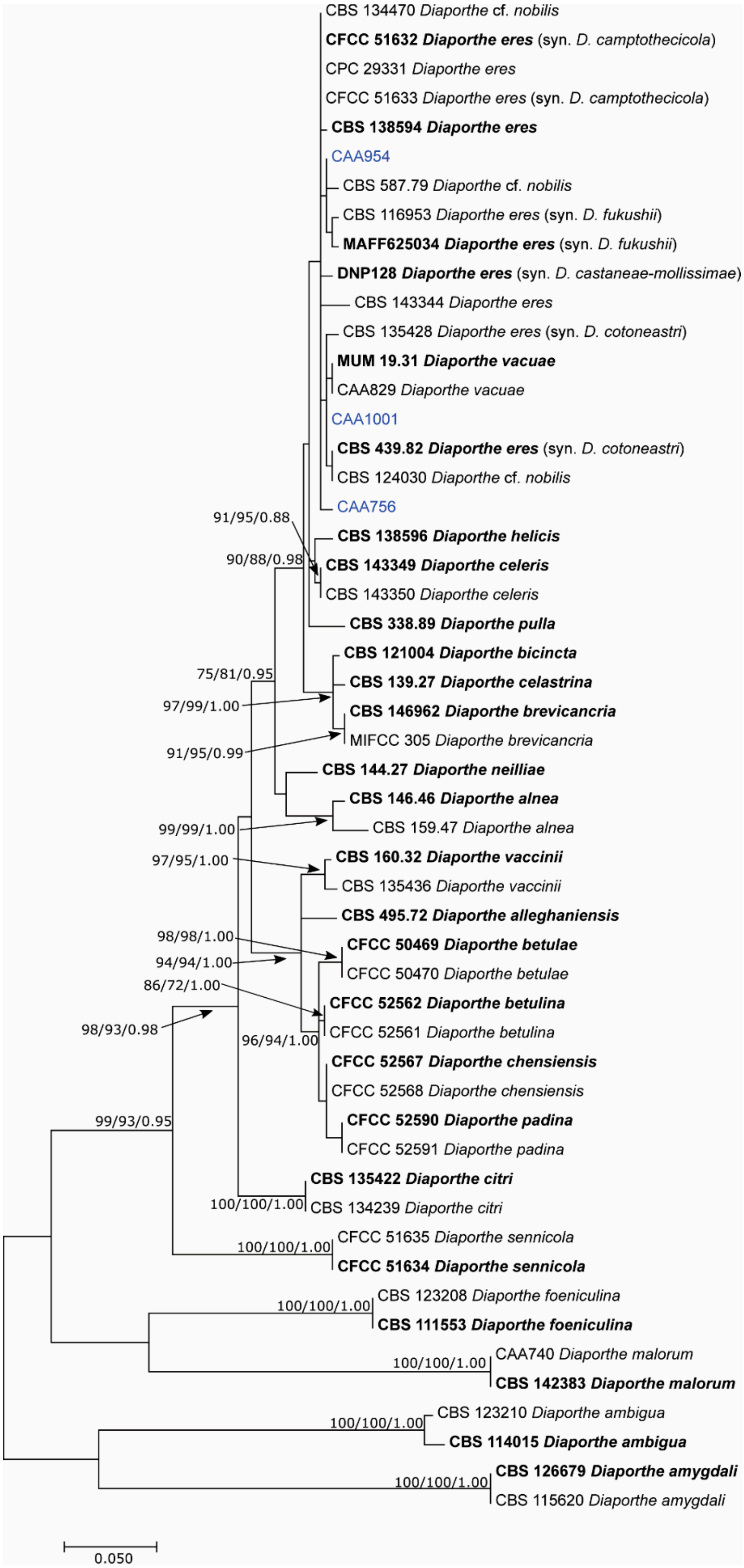

Figure S7. Maximum Likelihood tree based on TEF1-α sequence data for the Diaporthe eres species complex and related species. The ML tree was constructed based on the Hasegawa-Kishino-Yano model assuming uniform rates. Maximum Likelihood and Maximum Parsimony bootstrap values greater than 70% and posterior probabilities (PPs) inferred by Bayesian analysis greater than 0.70 are shown at the nodes. The ex-type strains are in bold. The isolates from this study are indicated in blue.

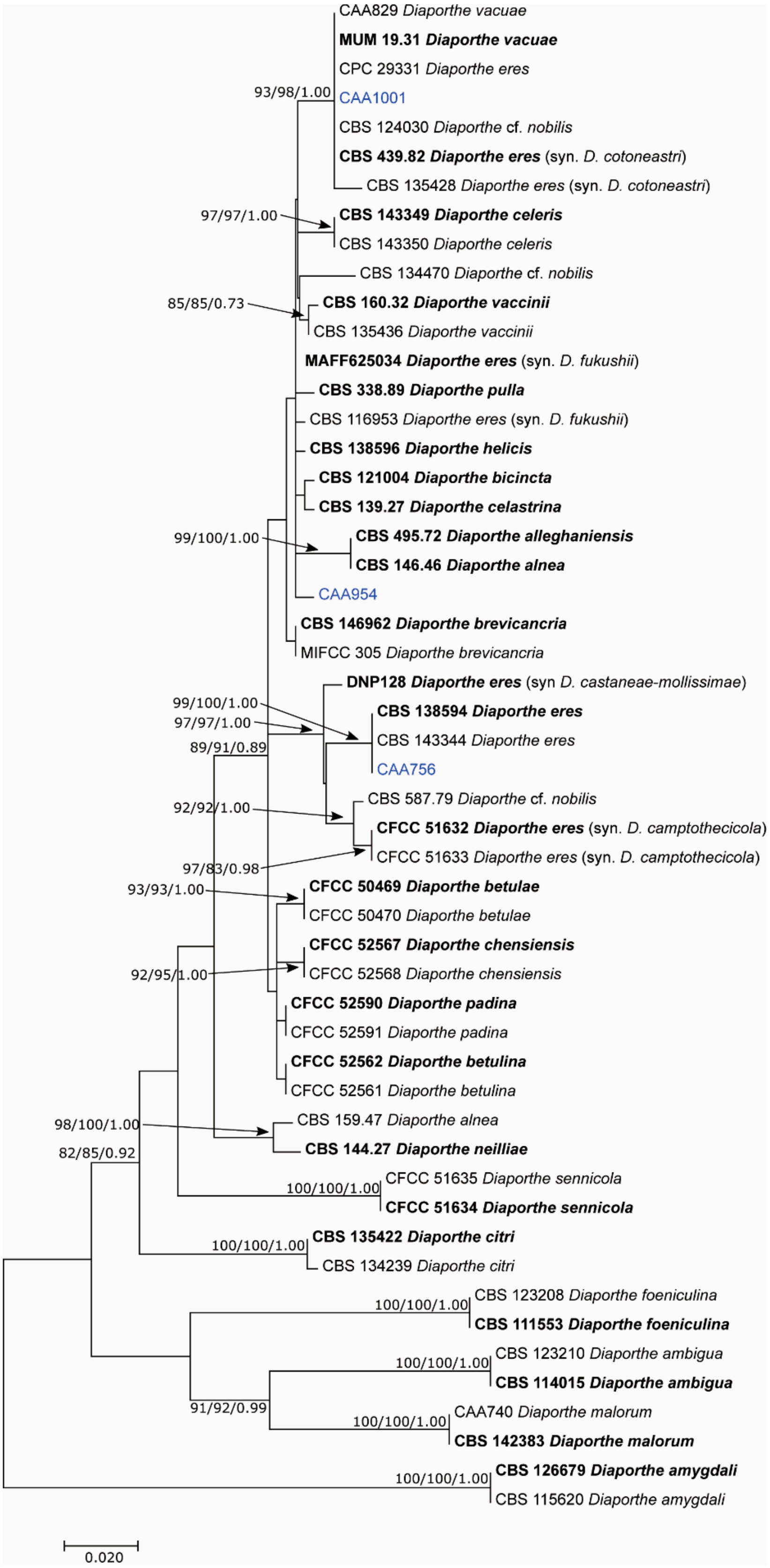

Figure S8. Maximum Likelihood tree based on TUB sequence data for the Diaporthe eres species complex and related species. The ML tree was constructed based on the Kimura 2-paramter model assuming a gamma distribution and invariant sites. Maximum Likelihood and Maximum Parsimony bootstrap values greater than 70% and posterior probabilities (PPs) inferred by Bayesian analysis greater than 0.70 are shown at the nodes. The ex-type strains are in bold. The isolates from this study are indicated in blue.

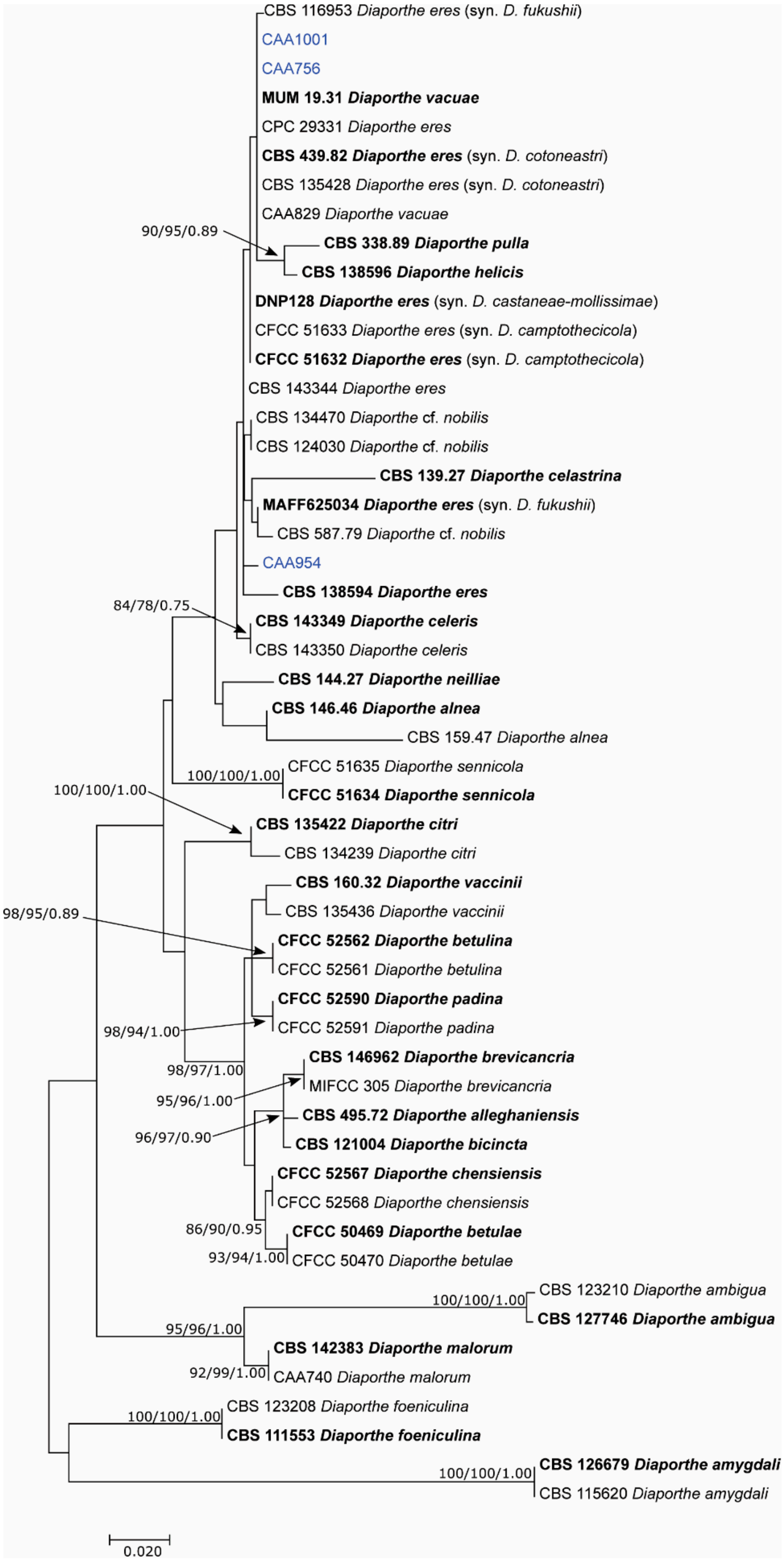

Figure S9. Maximum Likelihood tree based on HIS sequence data for the Diaporthe eres species complex and related species. The ML tree was constructed based on the Hasegawa-Kishino-Yano model assuming a gamma distribution and invariant sites. Maximum Likelihood and Maximum Parsimony bootstrap values greater than 70% and posterior probabilities (PPs) inferred by Bayesian analysis greater than 0.70 are shown at the nodes. The ex-type strains are in bold. The isolates from this study are indicated in blue.

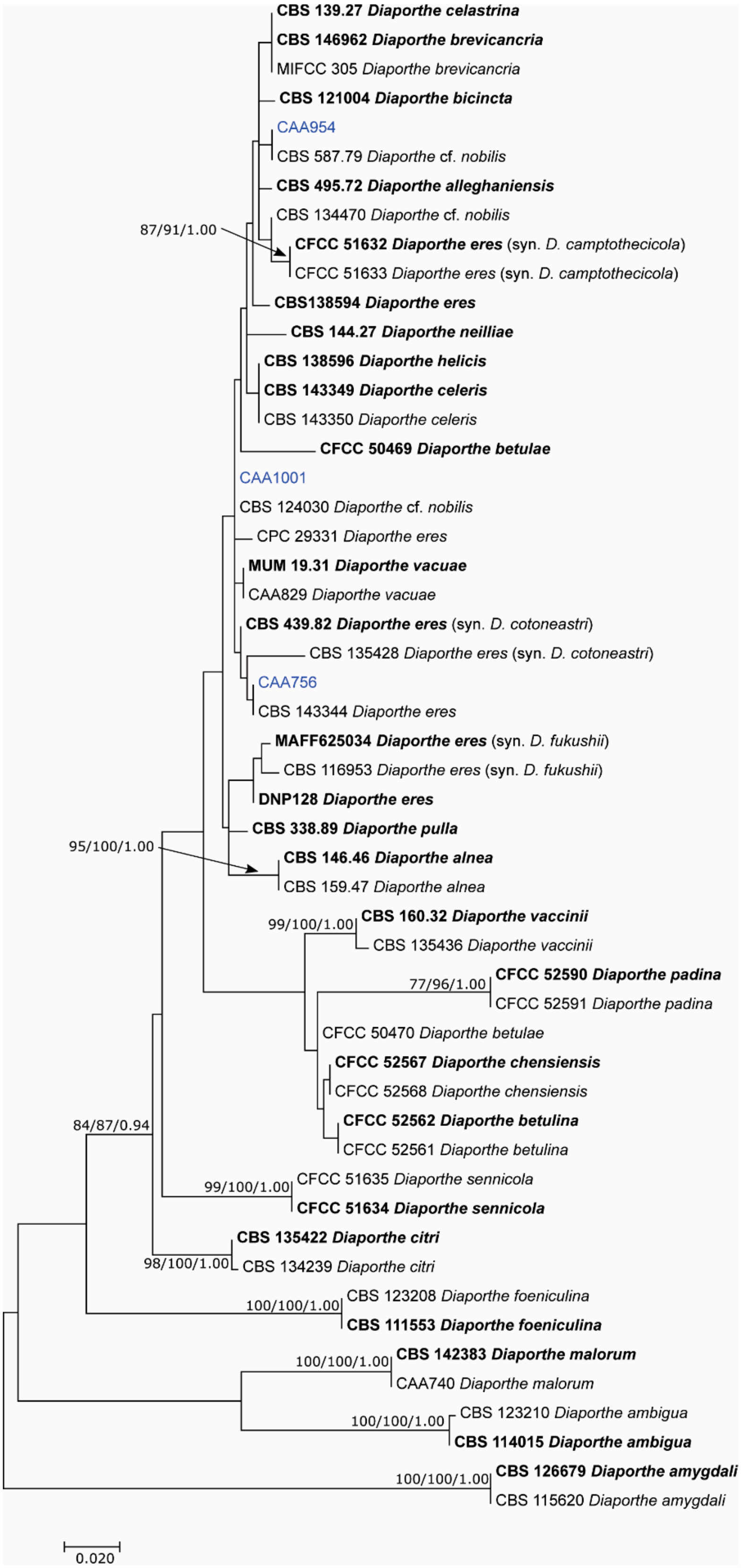

Figure S10. Maximum Likelihood tree based on CAL sequence data for the Diaporthe eres species complex and related species. The ML tree was constructed based on the Tamura 3-parameter model assuming a gamma distribution and invariant sites. Maximum Likelihood and Maximum Parsimony bootstrap values greater than 70% and posterior probabilities (PPs) inferred by Bayesian analysis greater than 0.70 are shown at the nodes. The ex-type strains are in bold. The isolates from this study are indicated in blue.

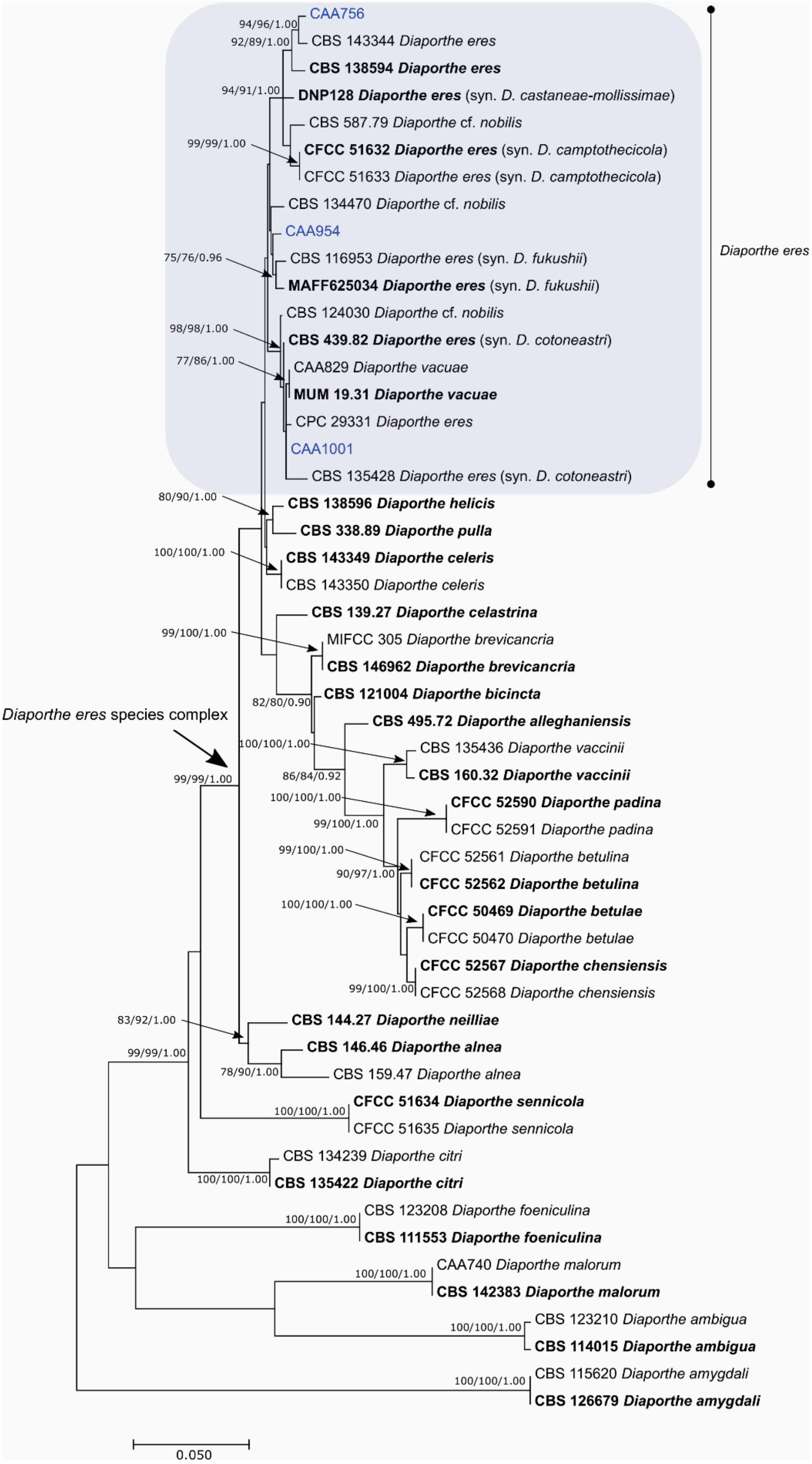

Figure S11. Maximum Likelihood tree based on TEF1-α, TUB, HIS and CAL sequence data for the Diaporthe eres species complex and related species. The ML tree was constructed based on the Kimura 2-paramter model assuming a gamma distribution and invariant sites. Maximum Likelihood and Maximum Parsimony bootstrap values greater than 70% and posterior probabilities (PPs) inferred by Bayesian analysis greater than 0.70 are shown at the nodes. The ex-type strains are in bold. The isolates from this study are indicated in blue.

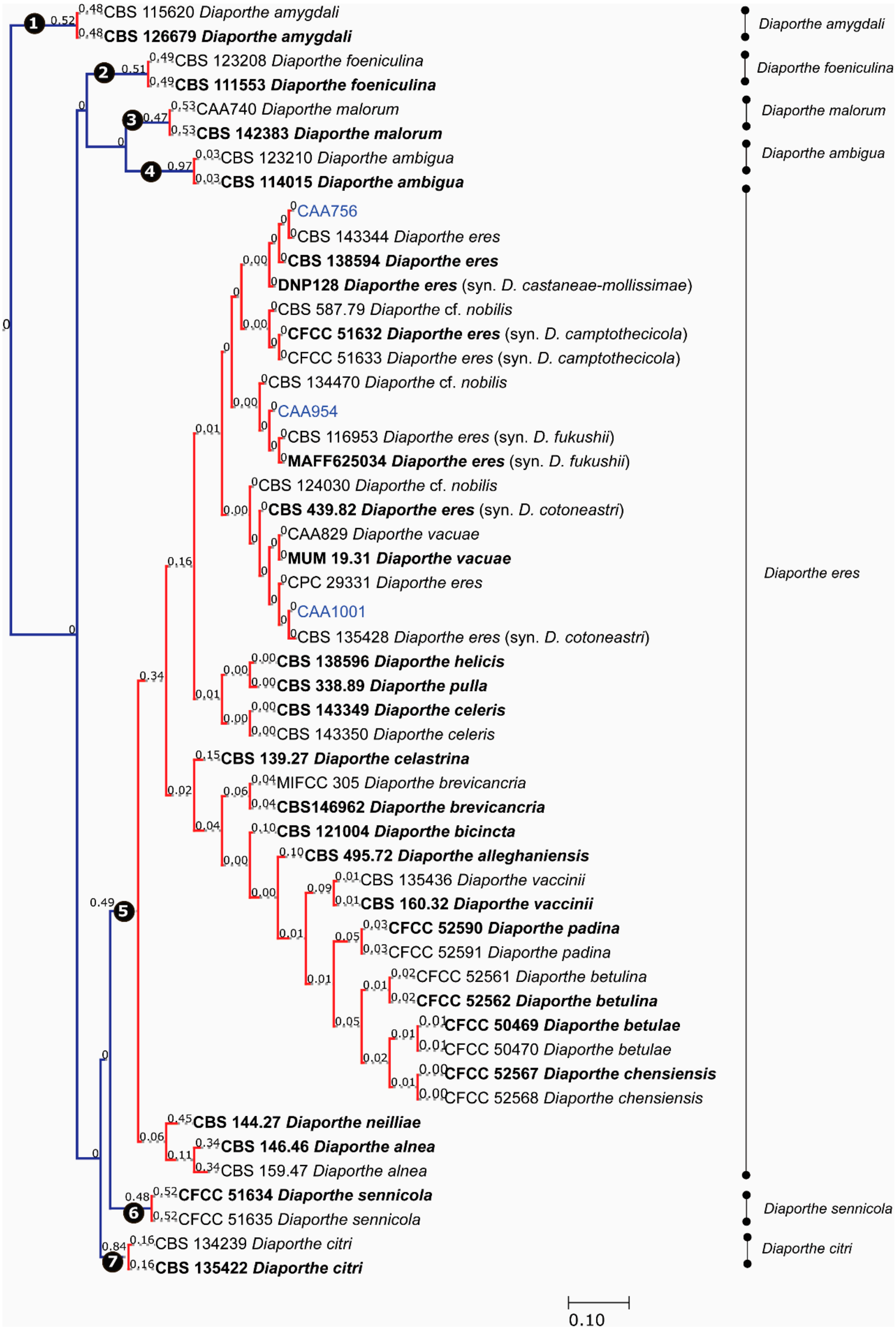

Figure S12. Results of the single PTP analyses for the Diaporthe eres species complex and related taxa, based on TEF1-α, TUB, HIS and CAL loci on Bayesian and Maximum Likelihood topologies. Putative species clusters are indicating using transitions between blue-colored to red-colored branches and represented by circles (1-7). The isolates obtained in this study are indicated in blue.
